# Multiomics Analyses of Two Sorghum Cultivars Reveal the Molecular Mechanism of Salt Tolerance

**DOI:** 10.3389/fpls.2022.886805

**Published:** 2022-05-23

**Authors:** Genzeng Ren, Puyuan Yang, Jianghui Cui, Yukun Gao, Congpei Yin, Yuzhe Bai, Dongting Zhao, Jinhua Chang

**Affiliations:** ^1^College of Agronomy, Hebei Agricultural University, Baoding, China; ^2^North China Key Laboratory for Germplasm Resources of Education Ministry, Hebei Agricultural University, Baoding, China

**Keywords:** sorghum, transcriptome, metabolome, proteome, flavonoids, salt stress

## Abstract

Sorghum [*Sorghum bicolor* (L.) Moench] is one of the most important cereal crops and contains many health-promoting substances. Sorghum has high tolerance to abiotic stress and contains a variety of flavonoids compounds. Flavonoids are produced by the phenylpropanoid pathway and performed a wide range of functions in plants resistance to biotic and abiotic stress. A multiomics analysis of two sorghum cultivars (HN and GZ) under different salt treatments time (0, 24, 48, and 72) was performed. A total of 45 genes, 58 secondary metabolites, and 246 proteins were recognized with significant differential abundances in different comparison models. The common differentially expressed genes (DEGs) were allocated to the “flavonoid biosynthesis” and “phenylpropanoid biosynthesis” pathways. The most enriched pathways of the common differentially accumulating metabolites (DAMs) were “flavonoid biosynthesis,” followed by “phenylpropanoid biosynthesis” and “arginine and proline metabolism.” The common differentially expressed proteins (DEPs) were mainly distributed in “phenylpropanoid biosynthesis,” “biosynthesis of cofactors,” and “RNA transport.” Furthermore, considerable differences were observed in the accumulation of low molecular weight nonenzymatic antioxidants and the activity of antioxidant enzymes. Collectively, the results of our study support the idea that flavonoid biological pathways may play an important physiological role in the ability of sorghum to withstand salt stress.

## Introduction

Salinity is one of abiotic stresses and it limits crop production (Xie et al., 2015). According to estimated statistics from UNESCO^1^ and FAO,^2^ in recent years, due to seawater indwelling and inadequate irrigation, over 7% of the world’s lands and 20% of irrigated lands are currently salt affected (Wang et al., 2003; Singh, 2021). Approximately half of cultivated fields will be affected by salinization by the middle of this century (Wang et al., 2004; Singh, 2021). Sodium salt, especially sodium chloride (NaCl), is the main substance that causes salt stress in the natural environment. Thus, one of the most important topics in crop stress resistance is to understand the resistance mechanism to salt stress, especially NaCl salt stress.

Excessive levels of Na^+^ and Cl^−^ ions affect the root system of plants firstly, impairing development in the short term due to osmotic stress (Munns and Tester, 2008). Long term and high levels of salt stress can disrupt the ability of plants to regulate ion homeostasis and also induce oxidative stress mediated by reactive oxygen species (ROS). Plant produced various peroxides, e.g., singlet oxygen (^1^O_2_), hydroxyl radical (HO•), hydrogen peroxide (H_2_O_2_), and superoxide radical (O_2_• ^−^) under salt stress. To escape the damage of stress, plants evolved variable adaptation mechanisms, such as the enhancement of metabolic adjustment, osmotic adjustment to compensate for osmotic stress. Non-enzymatic compatible solutes and antioxidants, such as proteins, phenolic compounds and flavonoids, and enzymatic antioxidants, such as peroxidase (POD), superoxide dismutase (SOD), catalase (CAT), ascorbate peroxidase (APX), played important roles in the ROS detoxification (Gupta and Huang, 2014; Ismail and Horie, 2017). As important secondary metabolites, flavonoids has been investigated extensively and were proofed having great role in response to abiotic stresses (Petrussa et al., 2013). The scavenging of free radicals by flavonoid molecules improves salt stress tolerance of plants (Yan et al., 2014; Chandran et al., 2019). For example, it has been reported that the accumulation of flavonoid could be induced by salt stress, and exogenous application of flavonoid could enhance salt tolerance in *Arabidopsis thaliana* (Chan and Lam, 2014). Similar results were obtained in *Ligustrum vulgare* (Agati et al., 2011). Bian et al. (2020) found that the HSFB2b, a class B heat shock factor, improves salt tolerance of *Glycine max* through the promotion of flavonoid accumulation (Bian et al., 2020). Kusano et al. (2011) compared the metabolic responses of wild-type *Arabidopsis* with that of mutants impaired in flavonoid and found that the accumulation of anthocyanin and tocopherol could improve the resistance to UV stress in *Arabidopsis* (Kusano et al., 2011).

Sorghum [*Sorghum bicolor* (L.) Moench] is the world’s fifth major cereal in terms of production and area harvested (FAOSTAT, 2019)^3^ and has been widely cultivated in more than 100 countries. Sorghum is not only an important staple food crop in the semi-arid tropics of Asia and Africa, but also an important source of forage and biofuel (Xie and Xu, 2019). Sorghum is widely grown in harsh environments because of its strong ability to resist drought and salt-alkali stress (Xie and Xu, 2019). Therefore, studying the salt tolerance mechanism of sorghum is essential for ensuring food security and promoting human health.

In recent years, multiomics analysis technology has been extensively utilized to examine abiotic stress. Transcriptomics, proteomics, and metabolomics technologies can enable more detailed monitoring of metabolic regulation and molecular processes to be implemented in plants grown in hostile environments (Masike et al., 2020; Li et al., 2020a; Hou et al., 2021). To examine how high salt content influences sorghum seedling development, we developed a research strategy whereby two conventional sorghum cultivars with contrasting salt sensitivity, the highly salt-tolerant landrace cultivar Gaoliangzhe (GZ) and hypersensitive improved cultivar Henong16 (HN), were initially cropped with a normal nutrient solution for 14 days and then the seedlings were placed under salt conditions for varying time periods. We performed a comprehensive omics analysis to reveal the striking changes in gene, metabolite, and protein pools. By comparing the transcriptome, metabolome, and proteome profiles of root tissues grown under salt stress, we identified specific genes, secondary metabolites, and protein complexes that are targeted for flavonoids and phenylalanine. Through omics technology analyses, we have obtained valuable information for understanding the salt tolerance mechanisms of sorghum and provided a better utilization for salt land.

## Materials and Methods

### Plant Materials and Growth Conditions

The seeds of two sorghum cultivars (HN and GZ) were chosen according to standard GB/T3543-1995 (Rules for agricultural seed testing-general directives)^4^ and disinfected with 75% ethanol, rinsed clean with deionized water, and cultivated in vermiculite for 5 days. Then, the uniform seedlings were transferred to hydroponic boxes and grown in an artificial climate chamber (ZRY-YY1000, Saifu, Ningbo, China). The growth conditions as follows: light/dark cycles: 14 h/10 h; light photosynthetic photon flux density: 1,000–1,200 μmol m^−2^ s^−1^; temperature: 28°C (light)/23°C (dark); relative humidity: 60 ± 5%. Every hydroponic box containing 2 L Hoagland’s solution [5 mM Ca(NO_3_)_2_.4H_2_O, 5 mM KNO_3_, 2 mM MgSO_4_.7H_2_O, 1 mM KH_2_PO_4_, 4.5 mM NH_4_HPO_4_, 100 μM EDTA-2Na, 100 μM FeSO_4_.7H_2_O, 4.5 μM H_3_BO_3_, 0.5 μM MnSO4, 0.1 μM CuSO4,ZnSO_4_.7H_2_O, 0.1 μM (NH_4_)_6_Mo_7_O_24_.4H_2_O] and the nutrient solution was changed once a day (Van Delden et al., 2020). When the sorghum seedlings grew to the three-leaf stage (~14 days), seedlings with even growth were carefully selected and divided into quarters, one for exposure to salt conditions (120 mM NaCl) and one for controlled conditions. Root tissue samples were harvested after 0 h (controlled conditions, referred to as C hereafter), 24 h (short-term treatment, referred to as S hereafter), 48 h (middle-time treatment, referred to as M hereafter), and 72 h (long-term treatment, referred to as L hereafter), immediately fixed with liquid nitrogen, and stored at −80°C for further analysis. The four treatments were harvested at same time. We selected two treatments (C and M) for transcriptome sequencing and RT-qPCR verification and chose four treatments (C, S, M, and L) for physiological parameters, proteome profiling, and metabolite detection. At least three independent biological replicates were used for each analysis (i.e., three for multiomics studies, and four for phenotypic and physiological characterizations studies; transcriptome sequencing uses three biological samples in RNA mixed sequencing). Five technical replicates of each treatment were conducted. Root lengths were measured after harvest.

### Phenotypic and Physiological Characterizations

Determination of antioxidant enzyme activities, including superoxide dismutase (SOD), peroxidase (POD), and malondialdehyde (MDA), were determined using the Superoxide Dismutase Activity Assay Kit, Peroxidase Activity Assay Kit, and Micro Malondialdehyde Assay Kit (Solarbio, Beijing, China), respectively. The tannin, total flavonoid (TF), and total phenol (TP) contents were detected with a Tannic Acid Content Assay Kit, Micro Plant Total Flavonoids Assay Kit, and Micro Plant Total Phenol Assay Kit (Solarbio, Beijing, China), respectively. The POD and SOD activities were determined by measuring the oxidation of 3,3′-dimethoxybenzidine and percentage of inhibition of pyrogallol autoxidation at 470 and 560 nm, respectively (Johansson and Håkan Borg, 1988; Doerge et al., 1997). The contents of tannins, TF, and TP were measured based on the tannic acid standard solution, the rutin acid standard solution, and gallic acid standard solution at 275, 470, and 760 nm, respectively (Wang et al., 2020; Palacios et al., 2021). The contents of MDA were measured at 450, 532, and 600 nm and calculated according to the manufacturer’s instructions (Spitz and Oberley, 1989). Statistical analysis was performed using IBM SPSS statistics for Windows, version 19.0 (SPSS, Chicago, IL, United States). Statistical significance tests were calculated using general Student’s *t*-test, and one-way analysis of variance (ANOVA) with Duncan’s multiple comparison test.

### Total RNA Isolation and Quantification

Total RNA was extracted using the Omini Plant RNA Kit (CWBIO, Beijing, China). The digestion of DNA was performed using DNase I. RNA purity and concentration were measured using a Nanodrop and Qubit 2.0 (Thermo Scientific, United States). RNA integrity was monitored on 1.2% agarose gels.

### RNA Library Construction and Data Analysis

The mRNA library was generated using the NEBNext Ultra™ RNA Library Prep Kit for Illumina (NEB, CA, United States). Briefly, the mRNA was purified using poly-T oligo-attached magnetic beads and fragmented using NEBNext First Strand Synthesis Reaction Buffer (NEB United States). First strand cDNA was synthesized using random hexamer primer and second strand cDNA was synthesized using RNase H and DNA Polymerase I. The double-stranded cDNA was purified by AMPure XP system (Beckman Coulter, Beverly, United States) after remaining overhangs converted into blunt ends and 3′ ends adenylation. Adapter ligation was carried out after adding “A” tail, and templates with the desired size range (200–250 bp) were subjected to PCR. Then the mRNA libraries were enriched by PCR and tested with Agilent 2100 and Qubit 2.0, and finally sequenced using Illumina HiSeq 2500 method. Raw data quality was controlled with criteria of less than 10% low-quality bases (Phred score < 20), followed by removing adapter sequences and of primers. Then, the clean reads were mapped to the sorghum reference genome (Sorghum_bicolor_NCBIv3)^5^ using TopHat2 software. Gene expression was quantified using the fragments per kilobase of transcript per million base pairs (FPKM) method. The differentially expressed genes (DEGs) were evaluated using DESeq R package (version 1.18.0; Ross Ihaka, University of Auckland, New Zealand) and screened with a fold change ≥2 and FDR ≤ 0.01.

### Reverse Transcription Quantitative PCR (RT-qPCR)

To validate the result of RNA-Sequencing, we selected eight up-regulated genes we are interested in after transcriptome analysis to further assess their expression patterns using RT-qPCR. The qualified RNA extraction was described in section the total RNA isolation and quantification. First-strand cDNA synthesis was conducted using a SuperRT cDNA Synthesis Kit (CWBIO, Beijing, China). RT-qPCR analysis was performed using a AugeGreen™ qPCR Master Mix (US EVERBRIGHT, Suzhou, China). The primers were designed by the NCBI primer-blast tool (Supplementary Table S2). The reaction was performed on a LightCycler 96 System (Roche, CA, United States) using the following protocol: predenaturation at 95°C for 5 min; 35 cycles of denaturation at 95°C for 15 s and renaturation at 60°C for 30 s; and extension at 72°C for 30 s. The β-actin gene (X79378) was used as an internal reference gene. The relative expression levels were calculated using the 2^−ΔΔCt^ method (Vastarelli et al., 2013). Each sample was performed in three replicates.

### Metabolite Extraction and Quantification

Secondary metabolite extraction and quantification were performed with the help of Wuhan MetWare Biotechnology Co., Ltd.^6^ following their standard procedures (Yuan et al., 2018; Zhang et al., 2019). Briefly, the freeze-dried roots were crushed using a grinder (MM 400, Retsch) at 30 Hz for 1.5 min. The powder (100 mg) was placed in 1.2 ml of 70% aqueous methanol at 4°C overnight and vortexed six times. The extracts were filtrated through a microporous membrane (0.22-μm pore size) after centrifuged at 12,000 *g* for 10 min. The sample extracts were analyzed using ultra-performance liquid chromatography–tandem mass spectrometry (UPLC-MS/MS) system. UPLC analysis was conducted with the SHIMADZU Nexera X2 instrument (Shimadzu, Kyoto, Japan) equipped with an Agilent SB-C18 column (1.8 μm, 2.1 mm × 100 mm). The mobile phase was consisted of pure water and acetonitrile (0.1% formic acid). The column temperature was 40°C. Gradient elution was performed at a flow rate of 0.35 ml min^−1^, with an injection volume of 4 μl for each sample. MS/MS analysis was completed with an Applied Biosystems 4500 Q TRAP system (Thermo, MA, United States). Quality control (QC) samples were inserted in every 10 test samples. Principal component analysis (PCA) and orthogonal partial least squares discriminant analyses (OPLS-DA) were performed. The differentially accumulating metabolites (DAPs) screened by combining the fold change and the variable importance in project (VIP) value. The metabolites with a fold change ≥1.5 or a fold change ≤0.67 and VIP ≥ 1 considered to the DAPs.

### Protein Extraction and Proteomics Analysis

Protein extraction and TMT-based proteomics analysis were conducted by Applied Protein Technology Co., Ltd.^7^ Briefly, sample (approximately 200 mg) lysis and protein extraction were performed using a sodium dodecyl sulfate (SDS)-DL-dithiothreitol (DTT) buffer (4% SDS, 100 mM Tris-HCl, 1 mM DTT, pH 7.6; Li et al., 2020b). 200 μg of protein was taken from each sample and digested using the filter-aided proteome preparation (FASP) method for trypsin digestion. Twenty microgram of protein for each sample were mixed with 5× loading buffer, respectively, and boiled for 5 min. The proteins were separated on 12.5% SDS-PAGE gel (constant current 14 mA, 90 min). Protein bands were visualized by Coomassie Blue R-250 staining. Next, 100 μg of peptide mixture was labeled with a tandem mass tag (TMT) reagent. TMT-labeled sample fractionation was conducted through strong cation exchange (SCX) resin chromatography using an AKTA purifier system. Liquid chromatography–tandem mass spectrometry (LC-MS/MS) analysis was performed using a Q Exactive hybrid quadrupole orbitrap mass spectrometer (Thermo Fisher, CA, United States). The raw data for each sample were searched using the MASCOT engine (Matrix Science, London, United Kingdom) embedded in Proteome Discoverer 1.4 software for identification and quantitation analysis.

### Bioinformatics Analysis

Gene Ontology (GO) annotation of the genes and proteins was implemented using Blast2GO software. The enrichment analysis of the DEGs, DEPs, and DAMs was determined by the GOseq R packages. The Kyoto Encyclopedia of Genes and Genomes (KEGG) annotation of the genes, proteins, and metabolites using a database^8^ mapped the pathways in the KEGG. KEGG pathway enrichment analysis was performed by KOBAS software. Subcellular localization of proteins was predicted by CELLO,^9^ which is a multi-class SVM classification system. Domain annotation of proteins was using the InterProScan software and identify protein domain signatures from the InterPro member database Pfam. Omics correlation analysis was performed using Metware cloud tools.^10^

## Results

### Phenotypic and Physiological Differences Between GZ and HN Under Salt Treatments

**Figure 1:**
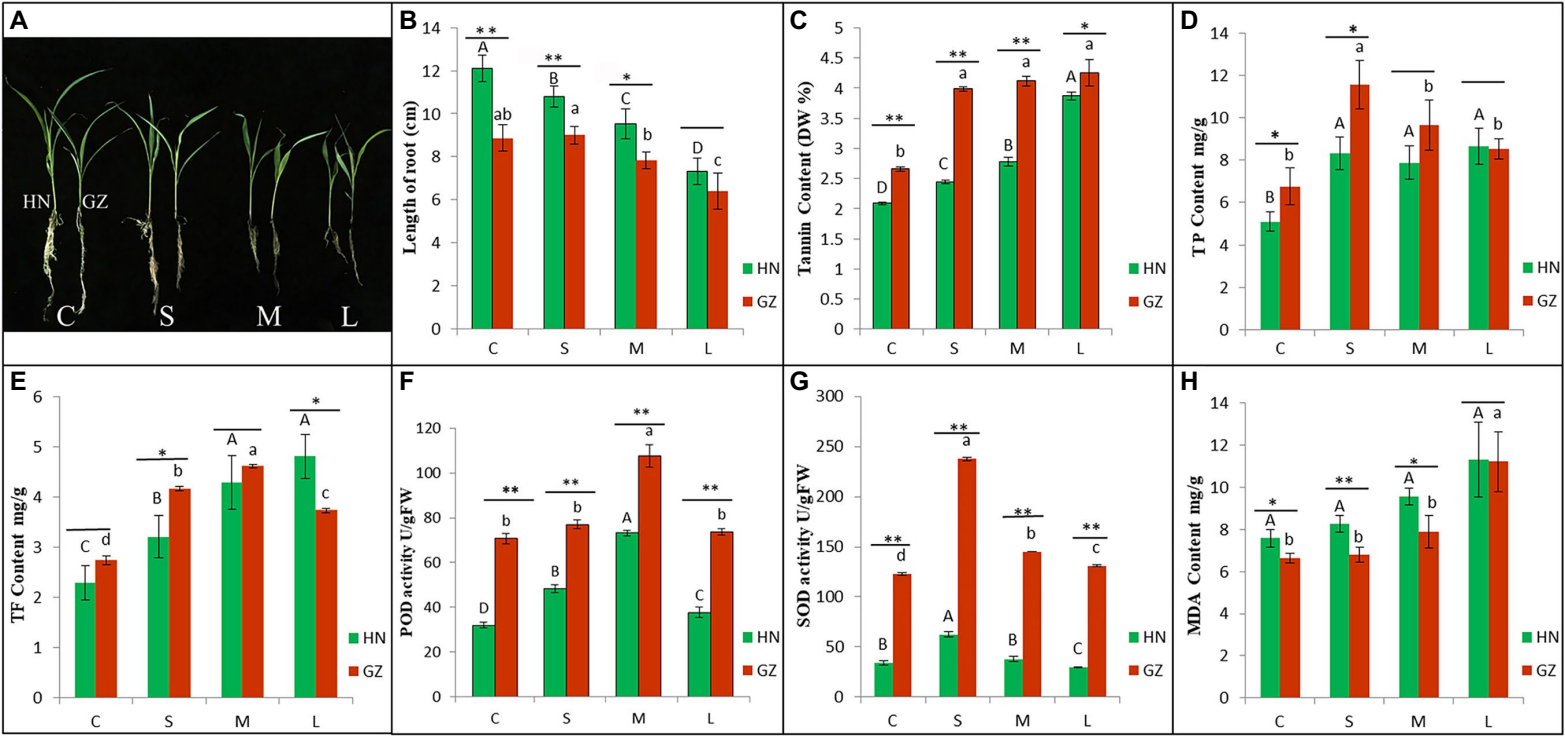
The differences of phenotypic and physiological between HN and GZ under salt treatments. **(A)** Phenotypes of Sorghum “GZ” and “HN” under salt stress. **(B)** Length of root. **(C)** Tannin content. **(D)** The total phenol (TP) content. **(E)** The total flavonoid (TF) content. **(F)** POD activity. **(G)** SOD activity. **(H)** MDA content. Error bars represent standard errors of three biological replicates. The statistical significance between varieties was determined by the Student’s *t* test (^**^p < 0.01,^*^p < 0.05). The statistical significance between treatments was evaluated by one-way analysis of variance (ANOVA) with Duncan’s multiple comparison test (*p* < 0.05). Capital letters indicate HN. Lowercase letters indicate GZ.

We analyzed the physiological and phenotypic characteristics of sorghum under salt stress. Under salt stress, the growth of the two sorghum varieties was inhibited to varying degrees (Figure 1A). The hypersensitive cultivar HN showed strong growth inhibition, but the highly tolerant cultivar GZ only displayed significantly accelerated root growth inhibition after 48 h (Figure 1B, Supplementary Table S1).

TP, TF, and tannin content in root of sorghum were significantly affected by salt stress. The tannin contents in GZ were significantly higher than HN. TP contents in GZ were significantly higher than HN excepted for M and L treatment. TF contents in GZ were significantly higher than HN excepted for C treatment (Figures 1C–E, Supplementary Table S1).

Peroxidase and SOD activity significantly increased with the increasing stress time and reach to the summit at M and S treatment, respectively, then decreased. MDA content significantly increased with the increasing stress time. POD and SOD activity of the highly tolerant cultivar GZ was significantly higher than that in the hypersensitive cultivar HN at four treatments (Figures 1F,G, Supplementary Table S1). MDA content in the highly salt-tolerant cultivar GZ was significantly lower than that in the salt hypersensitive cultivar HN under L treatment (Figure 1H, Supplementary Table S1).

### Transcriptome Differences Between GZ and HN Under Salt Treatments

We performed RNA-Sequencing, in order to investigate if the differential expression of genes affects the physiological and phenotypic differences under salt stress. Based on phenotypic and physiological trait analysis, 48 h (M) was the turning point at which the stress effect was significant in the two varieties (Figure 1B). Therefore, the samples of the two sorghum cultivars at 0 h (C) and 48 h (M) of salt stress treatment were selected for transcriptomic analysis.

**Figure 2:**
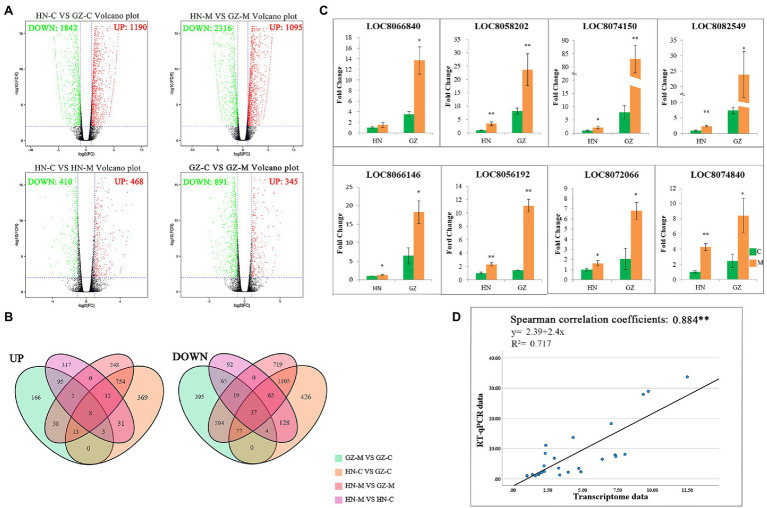
Transcriptome Differences between GZ and HN under salt treatments. **(A)** Volcano plot analysis on DEGs under control conditions and 48 h (M) salt treatments. The expressions of genes are showed by red spots (up-regulated), green spots (down-regulated). **(B)** Venn diagram of the DEGs under different salt treatments. UP: up-regulated, DOWN, down-regulated. **(C)** RT-qPCR of up-regulated expression genes. Error bars represent standard errors of three biological replicates. The statistical significance was determined by the Student’s *t* test (^**^p < 0.01; ^*^p < 0.05). **(D)** Linear regression between the levels of RT-qPCR data and transcript expression.

After quality control, we obtained a total of 25.07 Gb clean data with 84.98–85.07% of bases scoring Q30 and 53.02–53.93% of GC among the roots from the two cultivars grown with or without salt stress. The overwhelming majority of reads (75.37–77.42%) could be mapped to the sorghum reference genome, among which 72.12–74.49% were uniquely mapped (Supplementary Table S3). A total of 26,553 genes were functionally annotated in the databases and 841 new genes were found and enriched in the genomic information available in sorghum (Supplementary Table S4). Moreover, cross-comparisons (HN-C vs. GZ-C, HN-M vs. GZ-M, GZ-M vs. GZ-C, and HN-M vs. HN-C) identified 3,032, 3,411, 1,236, and 878 DEGs, respectively (Figure 2A). The top enriched KEGG terms contributed by these DEGs were ko00940 (phenylpropanoid biosynthesis), ko00360 (phenylalanine metabolism), ko00500 (starch and sucrose metabolism), and ko04075 (plant hormone signal transduction; Supplementary Figure S1).

**Figure 3:**
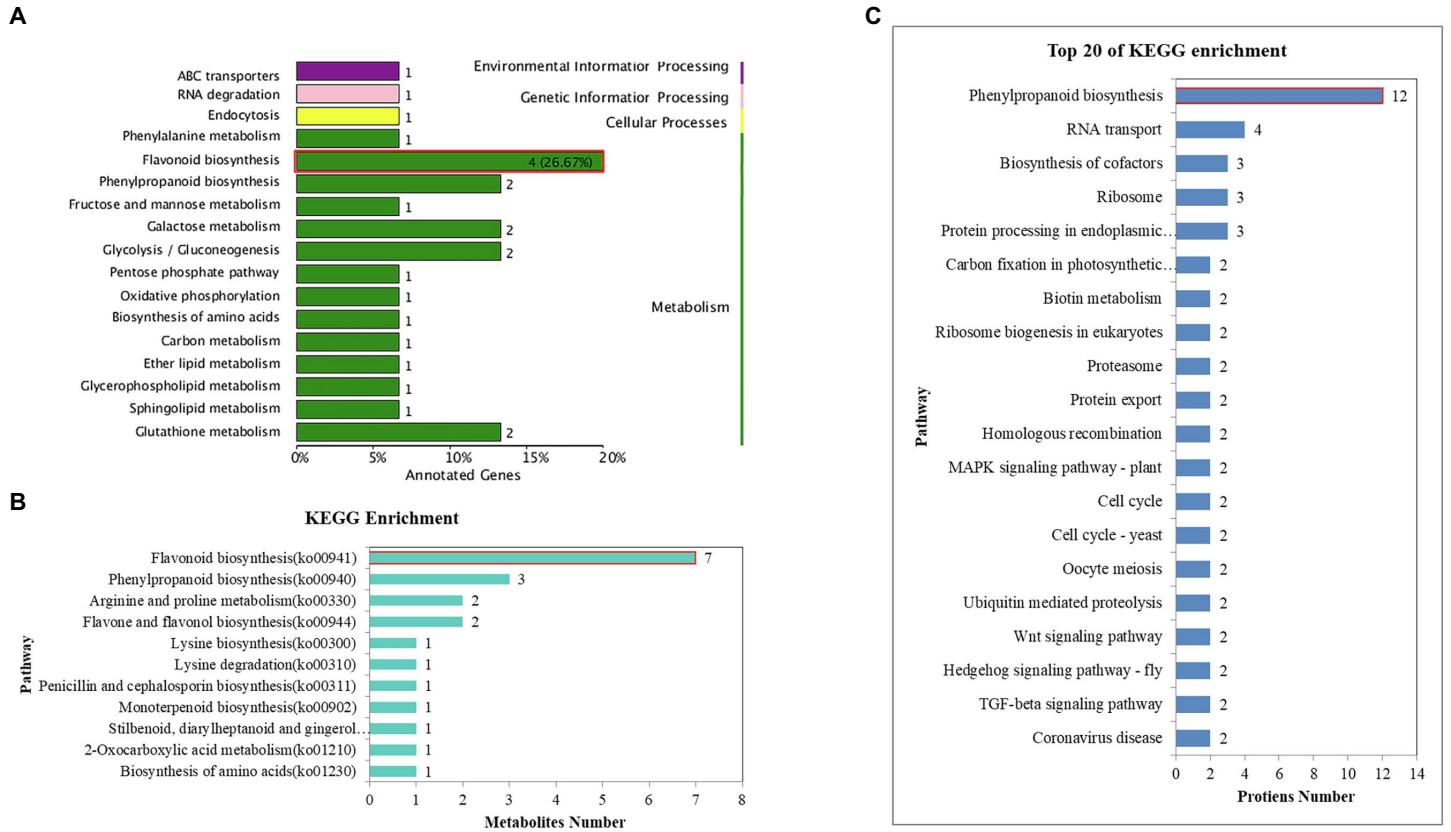
KEGG annotation and enrichment of the common DEGs, DAMs, and DEPs. **(A)** KEGG enrichment of the common DEGs. **(B)** KEGG annotation and enrichment of the common DAMs. **(C)** KEGG annotation and enrichment of the common DAPs.

Common DEGs were identified in the four comparisons to reduce the impact of time and/or circadian clock on salt stress. According to the Venn analysis, in total, 45 common DEGs were sustained, of which eight were upregulated and 37 were downregulated (Figure 2B). Based on the KEGG database, out of the eight upregulated common DEGs, four genes were allocated to the flavonoid biosynthesis pathway (ko00941; Figure 3A), encoding dihydroflavonol 4-reductase (DFR), leucoanthocyanidin dioxygenase (LDOX), anthocyanidin synthase (ANR), and flavonoid 3-hydroxylase (F3H).

To illustrate the correlation between gene expression and RNA-seq data with the salt stress response, the eight upregulated genes we interested in were selected to conduct RT-qPCR analysis. Detailed information on these genes is listed in Supplementary Table S2, and the RT-qPCR results are presented in Figure 2C. The results of RT-qPCR were in general agreement with those from the RNA-seq, with a Spearman correlation coefficient of 0.884, indicating that the transcriptome data were able to reflect transcript abundance in our study (Figure 2D). The elevated expression of the genes based on RNA-sequencing was higher in the highly salt-tolerant sorghum cultivar GZ than that in HN under salt stress by RT-qPCR, especially for the genes involved in flavonoid biosynthesis.

### Secondary Metabolites Differences Between GZ and HN Under Salt Treatments

**Figure 4:**
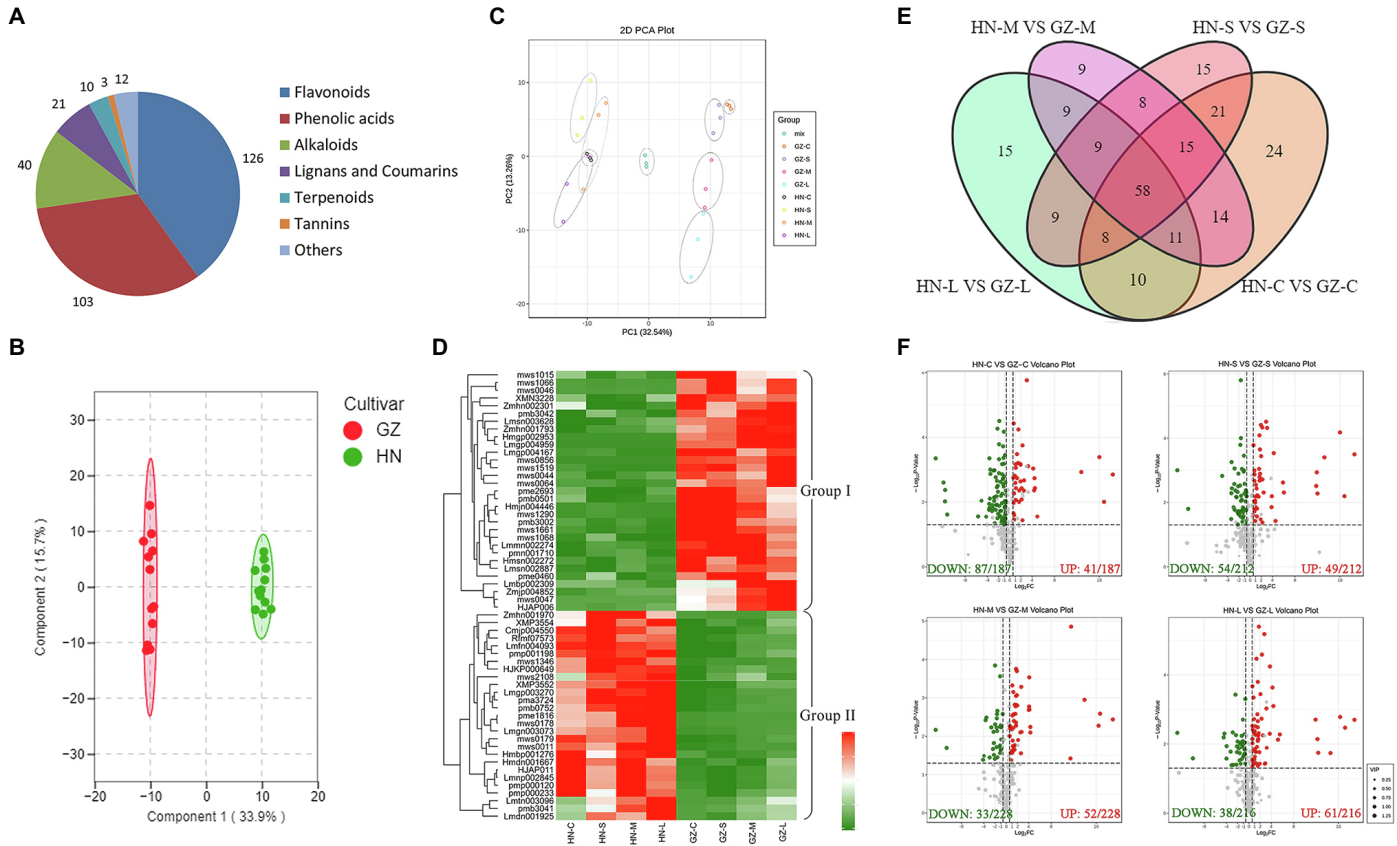
Metabolome differences between GZ and HN under salt treatments. **(A)** The classification of 315 secondary metabolites. **(B)** Principal component analysis (PCA) scores plot. **(C)** OPLS-DA score plot. **(D)** The heatmap of the common DAMs. **(E)** Venn diagram of the DAMs. **(F)** Volcano plot analysis on the DAMs under normal conditions and salt stress. Metabolite accumulation are represented by red spots (up-regulated), green spots (down-regulated).

Based on the transcriptome and physiological differences between the two sorghum cultivars, we expected that secondary metabolites, especially flavonoid compounds, may make an important contribution to sorghum salt resistance. To assess how secondary metabolites impact sorghum salt resistance, we conducted the metabolites analyses. We generated quantitative profiles of 315 secondary metabolites, of which 157 were identified in positive-ionization mode and 158 in negative-ionization mode in roots of salt tolerant (GZ) and sensitive (HN) cultivar under control and salt-treated environment. Among the 315 secondary metabolites identified, 150 metabolites were annotated from the Kyoto Encyclopedia of Genes and Genomes (KEGG).^11^ Total ion current (TIC) plots and multipeak detection plots of one QC sample are shown in Supplementary Figure S2. Overlay analysis for three QC samples and hierarchical heatmap clustering and correlation analysis for all samples were performed to evaluate the technical repeatability and reliability, respectively (Supplementary Figures S3, S4). We observed that the TIC plots had a perfect overlap, and all the biological replicates were grouped together, indicating good instrumental stability and repeatability. These metabolites were clustered into seven classes in terms of their contents. The most abundant class was flavonoids (126), followed by phenolic acids (103; Figure 4A). Detailed information on the 315 secondary metabolites is presented in Supplementary Table S5.

Combined analysis of three biological replicates, each containing root tissues, revealed substantial changes in the levels of numerous metabolites in HN and GZ seedlings. As expected, in the PCA score plot, two principal components (PC1 and PC2) were extracted to be 32.54 and 13.26%, respectively (Figure 4C). The PCA results showed that all samples presented as two groups, which revealed a clear separation between the two sorghum cultivars and a high similarity among the three biological replicates within each salt treatment, indicating that the experiment was reproducible and reliable. Interestingly, a clear separation of the stress treatments from the highly tolerant cultivar GZ was observed, in contrast to the sensitive cultivar HN, revealing that the two sorghum varieties responded to salt stress significantly differently at the metabolic level (Figure 4C). OPLS-DA was used to further discriminate the samples, and the metabolites of the GZ samples were obviously separated from those of the HN samples (*R*^2^*X* = 0.339, *R*^2^*Y* = 0.974, *Q*^2^ = 0.958; Figure 4B). The PCA and OPLS-DA results suggested that genetic variation strongly influenced the metabolite profiles of different sorghum varieties.

Pairwise analysis of metabolic differences under salt treatments was also performed. Hundreds of metabolites were shown to be significantly different between the two cultivars under the same treatment, in which there were 161 (44 upregulated and 117 downregulated), 143 (57 upregulated and 86 downregulated), 133 (75 upregulated and 58 downregulated), and 129 (74 upregulated and 55 downregulated) DAMs in HN-C vs. GZ-C, HN-S vs. GZ-S, HN-M vs. GZ-M, and HN-L vs. GZ-L, respectively (Figure 4F). The results of KEGG enrichment statistics showed that under same salt stress times were similar and a large number of the DAMs between the two cultivars enrichment in the five pathways: metabolic pathways, biosynthesis of secondary metabolites, flavonoid biosynthesis, phenylpropanoid biosynthesis, and flavone and flavonol biosynthesis (Supplementary Figure S5).

The Venn diagram depicted 59 common DAMs in the two cultivars under all stress conditions (Figure 4E). To further analyze the abundance profiles of metabolites under the different stress conditions, we performed hierarchical clustering of the common metabolites. This analysis resulted in two distinguishable groupings that revealed different metabolic patterns of the two cultivars under salt stress. Metabolites in Group I comprised 31 compounds (14 flavanols, seven phenolic acids, five dihydroflavones, two phenolamines, one lignan, one coumarin, and one alkaloid), which preferentially accumulated in the tolerant cultivar GZ. In contrast, Group II comprised 27 compounds (nine phenolic acids, six alkaloids, three flavonoids, two flavonoid carbonosides, two lignans, one coumarin, one chalcone, one monoterpenoid, one stilbene, and one tannin) accumulated mainly in the sensitive cultivar HN (Figure 4D). Further bioinformatic KEGG pathway analysis of the common DAMs revealed that the most prominent pathways included flavonoid biosynthesis, phenylpropanoid biosynthesis, and arginine and proline metabolism (Figure 3B). Seven metabolites (trihydroxyflavanone-rhamnosylglucoside, tetrahydroxyflavone, pentahydroxyflavanone, tetrahydroxyflavanol, caffeoylquinic acid, pentahydroxyflavan, and tetrahydroxyflavanone) were involved in the flavonoid biosynthesis process.

### Protein Differences Between GZ and HN Under Salt Treatments

To correlate the difference in transcriptome levels to secondary metabolite data, we analyzed the proteomic differences of the two sorghum cultivars at different salt treatment times. A total of 2,351,747 spectra (262,000 were the matched peptide spectrums), 60,119 peptides (50,635 were unique peptides), and 9,002 proteins (8,024 were quantified) were identified through LC-MS/MS identification and a search against the UniProt database employing MASCOT integrated with Proteome Discoverer 1.4 software, with 1% FDR by TMT quantification (Supplementary Table S6). The analysis results based on the peptide ion score distribution of the proteomic showed that ion score for 69.14% peptides surpassed 20 with 29.25 median score. Most of the peptides (>95%) were 5–23 amino acid residues long. In addition, almost 70% of the identified proteins corresponded to ≥2 peptides, suggesting that the MS data can be further analyzed. The smallest protein mass was just 3.61 kDa, while the largest reached 614.68 kDa. Most of the protein masses ranged from 10 to 60 kDa, accounting for 70.11% of all the identified proteins (Supplementary Figure S6). We performed subcellular localization analysis of these proteins. Out of the total proteins, 39, 28, and 22% proteins were located in the nuclear, cytoplasmic, and chloroplast, respectively. Other proteins were either localized at mitochondrial, plasma membrane or were extracellular (Supplementary Figure S7). A total of 7,527 proteins were annotated to the GO database and grouped into 52 functional groups, of which 22 functional groups were associated with biological processes, 15 with molecular functions, and 15 with cellular components. Among the GO classifications, “cellular process,” “metabolic process,” “binding,” “catalytic activity,” “cell,” “cell part,” and “organelle” were highly represented, with more than 3,000 proteins in each classification (Supplementary Figure S8).

**Figure 5:**
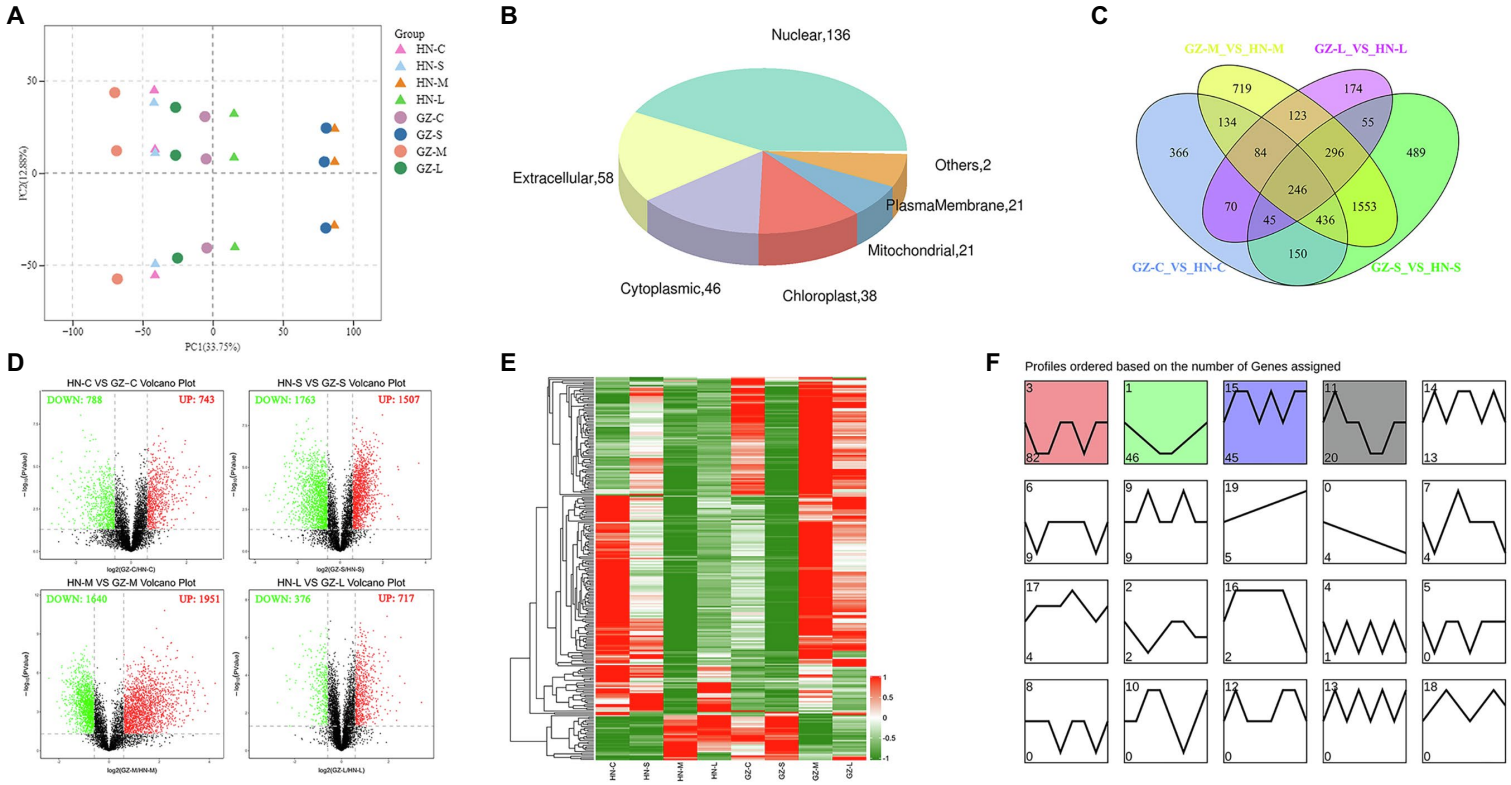
Proteome differences between GZ and HN under salt treatments. **(A)** Principal component analysis (PCA) analysis. **(B)** Subcellular localization analysis of the common DEPs. **(C)** Venn diagram of the DEPs. **(D)** Volcano plot analysis on the DEPs. Protein accumulation are represented by red spots (up-regulated), green spots (down-regulated). **(E)** The heatmap of the common DEPs. **(F)** Trend analysis shows of the common DEPs.

Similar to the metabolomic analysis, the proteomic PCA showed that genotype and salt stress had discernible effects, with HN being more severely impacted than GZ (Figure 5A). Furthermore, to identify the proteins most affected by salt stress, we selected those that were significantly impacted (fold change ≥1.5 or fold change ≤0.67 and FDR < 0.05). By pairwise comparisons at the same treatment time, C, S, M, L, in HN and GZ, we identified 1,261 (743 upregulated and 788 downregulated), 3,270 (1,507 upregulated and 1,763 downregulated), 3,591 (1,640 upregulated and 1,951 downregulated), and 1,093 (376 upregulated and 717 downregulated) DEPs, respectively (Figure 5D). Obviously, these results indicated that the proteomic effects of S and M treatment times were significantly greater than those of L treatment time. In the GO enrichment analysis. The DEPs were significantly enriched into 285, 352, 398, and 247 functional GO terms, respectively, of which 142,202,223,132 belonged to biological processes (BPs), 68, 80, 84, and 56 belonged to molecular functions (MFs), and 75, 70, 91, and 59 to cellular components (CCs), respectively (Supplementary Table S7). By using the Fisher’s Exact Test, we obtained the top 20 DEPs enriched GOs. We observed that the most significant GOs terms enriched in the BP, MF, and CC category were different (Supplementary Figure S9). KEGG enrichment analysis of DEPs from the same treatment time group showed enrichment of 86, 115, 114, and 74 pathways, respectively (Supplementary Table S8). It is worth noting that most of the significantly enriched KEGG pathways at different stress were similar and related to phenylpropanoid biosynthesis, ribosome, oxidative phosphorylation, protein processing in endoplasmic reticulum, and spliceosome (Supplementary Figure S10).

In total, according to the Venn analysis, a core set of 246 common DEPs that were significantly more or less abundant under the same treatments between the two varieties was obtained (Figure 5C). When the significantly common DEPs were illustrated by heatmaps, strong differences in GZ and HN tendencies were evident. Trend analysis shows that the accumulation of these common proteins in the two varieties is different (Figures 5E,F). The first cluster (including 82 proteins) showed downregulated stable-upregulated expression in HN but downregulated stable-regulated expression in GZ, and the second cluster (including 46 proteins) showed downregulated expression in HN and upregulated expression in GZ. We observed diverse subcellular localization of the common DEPs. Out of total 224 common DEPs, 136, 58, and 46 proteins were located in the nuclear, extracellular, and cytoplasmic, respectively. Other proteins were either localized at chloroplast, mitochondrial or were plasma membrane (Figure 5B). The result of the common DEPs domain annotation show that the most domain is peroxidase (Supplementary Figure S11). Protein functional analyses of the common DEPs were carried out using the GO and UniProt databases. The 246 common DEPs were classified into biological process (BP), cellular component (CC), and molecular function (MF) categories based on their functional features (Supplementary Figure S12). The major functional categories in the BP category were cellular process, metabolic process, single-organism process, and response to stimulus. For MF, binding and transporter activity were the most abundant groups. The cell, cell part, and organelle categories were the most abundant groups under CC. Most notably, by KEGG annotation, a total of 246 common DEPs were assigned to 40 pathways. These proteins were mainly distributed in phenylpropanoid biosynthesis, RNA transport, and biosynthesis of cofactors. The largest significantly enriched group was in phenylpropanoid biosynthesis, including 12 proteins (Figure 3C).

### Comprehensive Analysis of Transcriptome, Metabolome, and Proteome

**Figure 6:**
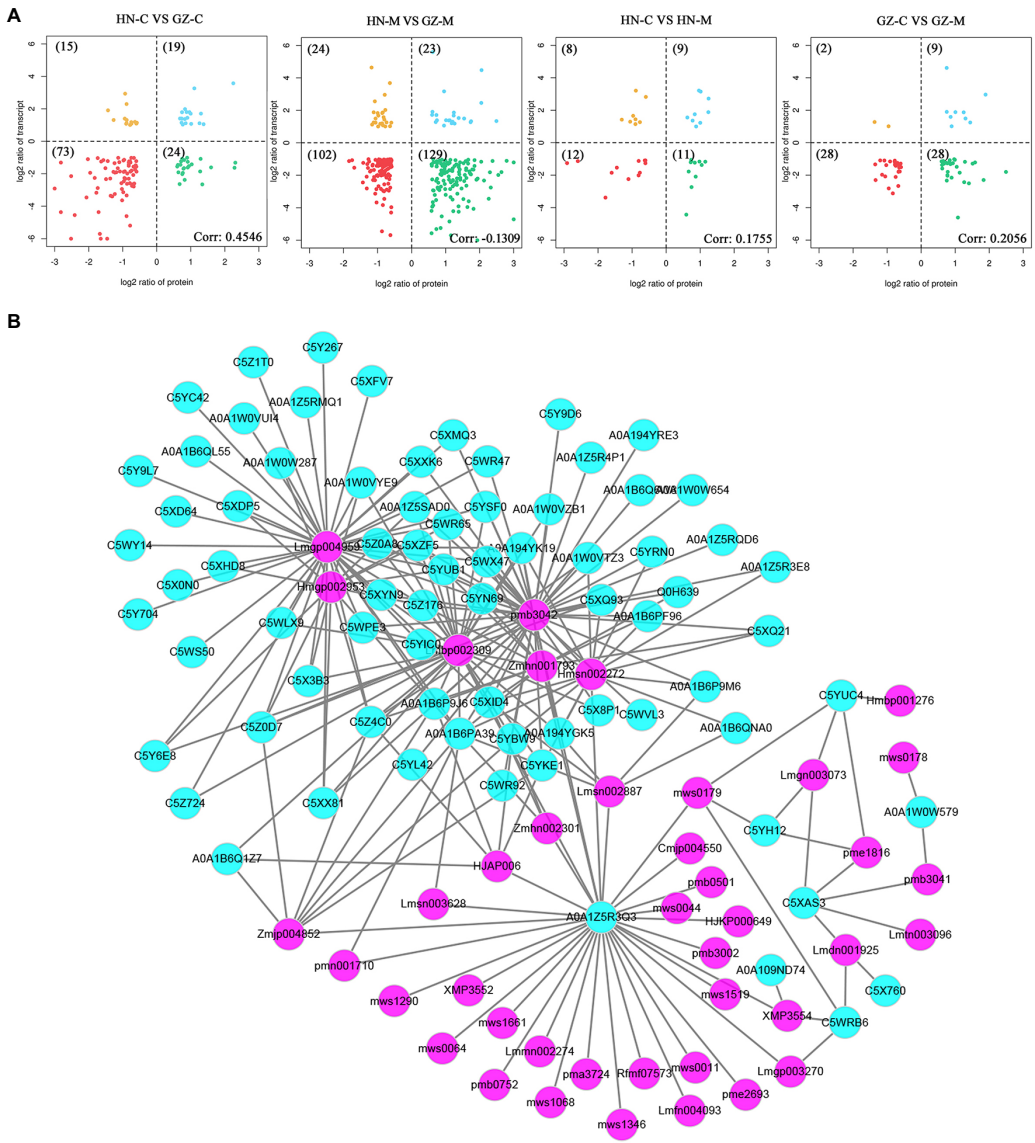
Multi-omics analyses of GZ and HN under salt treatments. **(A)** Four-quadrant diagram analysis between the common DEGs and the common DEPs in sorghum in response to salt. **(B)** Correlation network constructed with the common DEPs (green) and the common DAMs (purple) under salt stress (*R*^2^ > 0.7).

The results of transcriptome, proteomics, and metabolome analysis showed the differentiation of genes, proteins, and secondary metabolites in the phenylpropanoid pathway and flavonoid biosynthesis pathway between the different sorghum cultivars. In the transcriptome analysis, the genes regulating the biosynthesis of flavonoids were upregulated. Similar to the proteomics and metabolome analyses, an obvious separation between the two different samples was observed, of which the largest group was related to phenylpropanoids and flavonoids. As shown in Figure 6A, the common DEPs showed a positive correlation for the common DEGs of HN-C vs. GZ-C, HN-C vs. HN-M, GZ-C vs. GZ-M, and a negative correlation with HN-M vs. GZ-M, implying that salt stress may have different effects on proteins and genes. This result is similar to the results obtained above.

The metabolism and protein regulation network in plants is very complex. One of the important ways for proteins/metabolites to function is to interact with other proteins/metabolites. Highly correlated proteins/metabolites may have similar functions and may be the key factor affecting metabolism or signal transduction. Therefore, the study of protein–metabolite correlations is of great significance. Pearson correlation coefficients for common DEPs and common DAMs were calculated to reveal the synergistic interaction between the proteome and metabolome and identify more important factors. The results show that there are complex correlations between the common DEPs and the common DAMs. Sixty-five common DEPs were strongly correlated (*R*^2^ > 0.7 and *p* < 0.05), with 39 common DAMs under salt stress (Figure 6B). The correlation between metabolites and proteins does not have a one-to-one correspondence. For example, the protein A0A1Z5R3Q3 (LOC8061086) was significantly correlated with the relative content of 31 common DAMs. The top five highly correlated metabolites were chrysoeriol-glucoside, dihydroquercetin, demethyl coniferin, caffeoyl xylose, and p-Coumaric acid-4-O-glucoside. There was also a significant correlation between naringenin (Lmgp004959) and 41 common DEPs. The top five proteins were C5X3B3, C5Z176, C5Z4C0, C5Y6E8, C5YUB1, encoded by LOC8073079, LOC8072721, LOC8069161, LOC8068289, and LOC8065892, respectively.

## Discussion

### The Different Effect of Salt Stress on Phenotypic and Physiological at Seedling Stage in Two Sorghum Cultivars

In general, the germination and seedling stage is the weakest period of plant to abiotic stress. Therefore, the germination and seedling stage is crucial for the establishment of plants (Song et al., 2008; Vennapusa et al., 2021). Salt stress has serious influence on root length, especially for hypersensitive improved cultivars (Ukwatta et al., 2021). It is similar to the results of our current study (Figure 1B). ROS are generated by plants under high exogenous salt concentration. As key secondary messengers, ROS can trigger subsequent defensive measures. To overcome salt-mediated oxidative stress, plants have developed a comprehensive and intricate system for scavenging high levels of ROS by using antioxidant enzymes (Dietz et al., 2016). The correlation between salt tolerance and antioxidant capacity has been found in wheat (Meneguzzo et al., 1999). In the present study, the antioxidant enzyme activities under salt conditions were significantly higher than those under the control treatment, and the antioxidant enzyme activities in GZ increased faster than that in HN (Figures 1F,G). Similar results have been also observed in spring wheat previously (Raza et al., 2007). On the other hand, abiotic stress leads to the upregulated expression of polyphenol compounds, which promotes the antioxidant capacities of plants (Hodaei et al., 2018). In maize, the accumulation of tannin, TP, and TF in salt-tolerant varieties were higher than that in salt-sensitive varieties under salt stress (Hichem et al., 2009). The same phenomenon was observed in the present study (Figures 1C–E). A more rapid and elevated accumulation of flavones was observed in the resistant cultivar GZ than in the susceptible cultivar HN.

### Comparison of Different Omics Studies on Sorghum Under Salt Stress Condition

In recent years, omics technologies have been widely utilized by researchers in the field of sorghum abiotic stress especially salt stress (Surender Reddy et al., 2015; Punia et al., 2020). The experimental designs and outcomes of our study showed differences as well as similarities from previous studies. In the previous studies (Ma et al., 2020; Sun et al., 2020), integrated transcriptomic and metabolomic and transcriptomics techniques were used, respectively. In our work, three omics techniques were performed, to detect the differences in two different salt tolerance sorghum cultivars under salt stress, which help us improve the understanding of the biosynthetic networks more comprehensively (Zhou et al., 2019).

Transcriptomic analysis provides a fast way to find different expression genes in the plant response to abiotic stress. Some genes, as key components, were the primary factors affecting the signal transduction pathways in response to stress (Fujita et al., 2006). Hundreds of genes inducible by drought, cold, and high salinity have been identified in *Arabidopsis* using transcriptomic analysis (Seki et al., 2002). Our previous studies also explored dozens of DEGs related to salt tolerance in other sorghum genotypes planted in salt environments based on transcriptomic technology (Cui et al., 2018). Although the salt stress condition is different, the outcomes of our study showed similarities from previous studies by Ma et al. (2020). Ma et al. (2020) found that anthocyanin biosynthesis-related genes such as LOC8074150 which editing anthocyanidin synthase were up-regulated under moderate salt−alkali stress. In our work, the comparative analysis using RNA-Sequence revealed that a lot of the DEGs were located in the flavonoid biosynthesis pathway, indicating flavonoid may potentially confer protection against salt stress (Figure 3A).

The biosynthesis of flavonoids starts with phenylalanine and involves a series of enzymatic reactions. CHS, CHI, F3H, F3’H or F3’5’H, DFR and ANR are the key enzymes in this pathway. Our results of transcriptome analyses showed that the majority of the common DEGs were distributed to flavonoids biosynthesis and conducted the synthesis of key enzymes including ANR, DFR, and F3H. *F3H* genes have been cloned and characterized from a variety of plant species (Arnone et al., 1992; Charrier et al., 1995; Si et al., 2022). Overexpression of *F3H* from *Pohlia nutans* increased the tolerance of *A. thaliana* to salt stresses (Li et al., 2017). Liu et al. (2013) also previously reported that *F3H* gene is the key regulator of flavonoid biosynthesis that participates in the responses to UV stress and salinity (Liu et al., 2013). Kim et al. (2017) previously reported that *DFR* gene can be effectively manipulated to modulate salt and drought stress tolerance in *Brassica napus* L.

**Figure 7:**
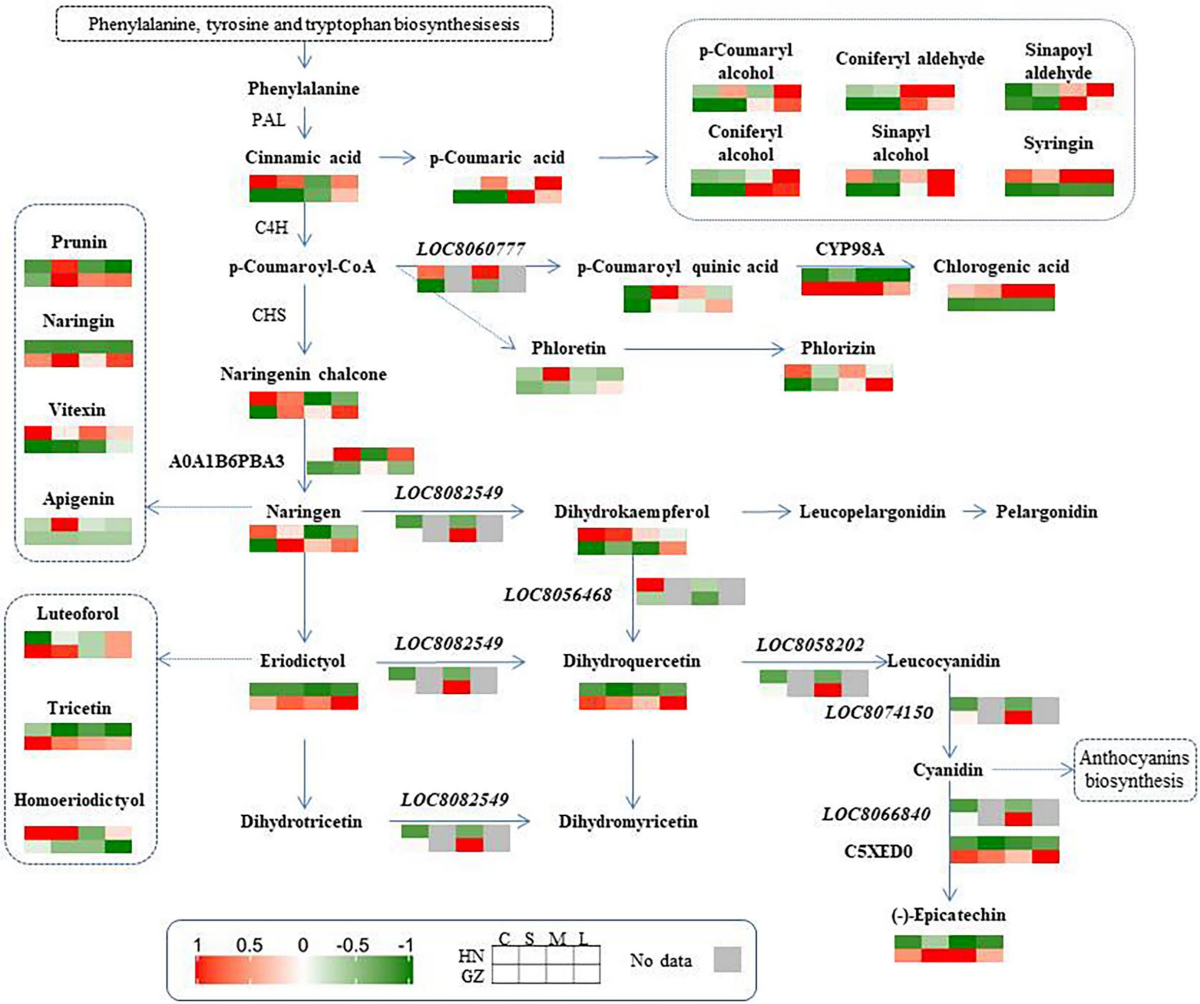
Phenylpropanoid and Flavonoid biosynthesis pathway. Genes, proteins, and metabolites with differential abundance involved in phenylpropanoid and flavonoid biosynthesis metabolism were mapped to the corresponding metabolic pathways in KEGG.

Metabolism makes an important contribution to the regulation mechanisms under abiotic stress because plants have extremely rich and variable metabolic profiles (Li et al., 2021). Some metabolites are vital for signaling and adaptation to environmental stress (Yang et al., 2020). As one of the most important secondary metabolites, flavonoids are widely distributed in plants (Tanaka et al., 2010). Studies on several plant species have demonstrated that the accumulation of flavonoids is critical for survival under difficult growth conditions (Liu et al., 2012; Petrussa et al., 2013; Chen et al., 2017). Increasing the levels of flavonoids by transgenes UDP-sugar glycosyltransferases (UGTs) into *Arabidopsis* can increase salt and drought stress tolerance (Zhang et al., 2021). It has been reported that the sorghum variety with high pathogen resistance showed a more rapid and elevated accumulation of flavonoids than the susceptible cultivar after inoculation with the anthracnose pathogen (Du et al., 2010). In our study, the sorghum-resistant cultivar showed a more rapid and elevated accumulation of flavonoids than the susceptible cultivar after salt stress. Out of the seven common DAMs involved in the flavonoid biosynthesis process, the levels of five metabolite accumulations were higher in the highly tolerant cultivar GZ, including trihydroxyflavanone-rhamnosylglucoside, tetrahydroxyflavone, dihydroquercetin, pentahydroflavan, and tetrahydroxyflavanone (Figures 3B, 7). Previous studies show that pentahydroxyflavan on the outer surface of the chloroplast envelope might additionally quench ROS formed outside the chloroplast (Mullineaux and Karpinski, 2002). The C-glycosyl flavone maysin in silk tissues is responsible for *maize* insecticidal activity toward corn earworms (Rector et al., 2002). Flavones also serve as antioxidants to protect *Arabidopsis* from UV irradiation (Bieza and Lois, 2001). In our work, the TF and TP contents in the two cultivars were affected by salt. These results indicated that there were links between sorghum salt resistance and the flavonoid pathways.

Plant resist to environment stress is a complex pathway, including transcriptome, metabolome, and proteomic. Proteomics is a powerful tool and can provide more qualitative and quantitative information about responses to abiotic stress. A study in sorghum leaves using comparative proteomics identified protein groups responding to salt stress (Swami et al., 2011). In our research, in contrast to the transcriptome sequencing and metabolome results, the proteomic sequencing results showed that most of the differential proteins were allocated to the phenylalanine pathway (Figure 3C). The result is partial consistency with the transcriptome and metabolism. Interestingly, most of the DEPs participated in the phenylalanine pathway are the enzymes of flavonoid or flavone biosynthesis in our result. Peroxidase (A0A109ND74, E1.11.1.7) catalyzes the oxidation of phenylpropanoids to their phenoxyl radicals. In this study, peroxidase induced by salt stress and the expression of peroxidase in GZ is higher than that in HN. In addition, glutathione S-transferase (C5WY74 EC:2.5.1.18) is one of the “core” enzymes acting in the flavone and flavonol biosynthesis. Glutathione S-transferase were induced in GZ and degraded in HN in our study. Previous research indicated that overexpression of the glutathione S-transferase could enhanced salt resistance of mulberry (Gan et al., 2021).

In plants, the amino acid phenylalanine is a substrate of both primary and secondary metabolic pathways (Peng et al., 2018). Phenylalanine is the precursor of flavonoid, phenol, and anthocyanin synthesis, which plays a crucial role in the plant response to biotic and abiotic stress (Agati et al., 2013). Previous research indicated that the pathways associated with phenylalanine have important functions in the plant response to environmental stress (Savoi et al., 2016). The proteomics analysis in our experiment linked the DEGs and DAMs and enhanced the understanding of flavonoid function in sorghum resistance to salt.

### In-depth Multiomics Analysis to Identify Possible Salt Tolerance Pathway of Sorghum

Our in-depth omics analysis also provides a feasibility to map how sorghum seedlings are impacted by salt stress at the transcriptional and posttranscriptional levels. Based on the flavonoid pathway and the phenylalanine pathway downloaded by the KEGG, three proteins, six genes, and 25 secondary metabolites were mapped (Figure 7). Four genes (LOC8058202, LOC8074150, LOC8066840, and LOC8082549) were upregulated at the two cultivars under salt stress. In contrast, two genes (LOC8060777 and LOC8056468) were upregulated only at HN. The contents of seven metabolites (trihydroxyflavanone-rhamnosylglucoside, dihydroquercetin, eriodictyol, tricetin, naringenin-glucoside, epicatechin, and pentahydroxyflavan) in the salt-tolerant cultivar were higher than those in the hypersensitive cultivar. For proteins, two proteins (CYP98A and C5XED0) were upregulated in GZ under salt stress.

In sorghum, the abundance of some flavonoid metabolites is significantly related to the expression of flavonoid biosynthesis genes (Ma et al., 2020). Similar to the current study, flavonoids varied with the degree of stress and genotype, and the dynamic changes in flavonoid content at different genotypes were of great significance, particularly in the highly tolerant cultivar. The genes, proteins, and metabolites involved in this pathway were obviously different between highly tolerant and sensitive cultivars, and coordinated variations were detected. For example, it is noteworthy that the abundance of epicatechin was significantly correlated with the expression of the anthocyanidin reductase (EC 1.3.1.77) gene and its coding protein (C5XED0; Figure 7).

These results provide us with references for further studying the deep mechanisms of the molecular network in response to salinity stress. Combining phenotype and multiomics analysis considerably improves our knowledge of the molecular mechanisms and pathways underlying the response of sorghum to salt stress and provides important clues on how to relieve salt stress during the seeding stage.

## Conclusion

The results of the current study showed that salt stress led to differentially regulated expression of genes as well as significant variations in proteins and secondary metabolites. We identified four key genes and seven key secondary metabolites enriched in the flavonoid biosynthesis pathway. We also identified 12 key proteins enriched in the phenylpropanoid biosynthesis pathway. By the multiomics analyses, we found that flavonoid biosynthesis pathway plays important role in sorghum resistance to salt stress and the gene LOC8066840 which is responsible for protein C5XED0 and epicatechin biosynthesis may be the key gene in regulating the ability of sorghum to withstand salt stress. The results of our study provide insights for further understanding the molecular mechanism of salt tolerance in sorghum, and lay the foundation for exploring and cloning salt-resistant gene and genetic improvement of sorghum. Meanwhile, this work can also provide reference to other crops in salt resistance improving.

## Data Availability Statement

The datasets presented in this study can be found in online repositories. The names of the repository/repositories and accession number(s) can be found at: The raw data files for this RNA-seq during our experiment are deposited in NCBI (BioProject: PRJNA395348 and PRJNA816817). The mass spectrometry proteomics data have been deposited to the ProteomeXchange Consortium (http://proteomecentral.proteomexchange.org) *via* the iProX partner repository with the dataset identifier PXD032125.

## Author Contributions

GR and PY: roles/writing—original draft and investigation. CY and YG: supervision. YB and DZ: software. JCu and JCh: methodology, project administration, and writing—review and editing. All authors contributed to the article and approved the submitted version.

## Funding

This work was supported by the National Key R&D Program of China (2019YFD1000700 and 2019YFD1000703) and the Hebei Key Research & Development Program (20326347D and 21326305D).

## Conflict of Interest

The authors declare that the research was conducted in the absence of any commercial or financial relationships that could be construed as a potential conflict of interest.

## Publisher’s Note

All claims expressed in this article are solely those of the authors and do not necessarily represent those of their affiliated organizations, or those of the publisher, the editors and the reviewers. Any product that may be evaluated in this article, or claim that may be made by its manufacturer, is not guaranteed or endorsed by the publisher.

## Footnotes

^1^ https://www.unesco.org/

^2^ https://www.fao.org/

^3^ https://www.fao.org/faostat/

^4^ http://openstd.samr.gov.cn/bzgk/gb/index

^5^ https://plants.ensembl.org/Sorghum_bicolor

^6^ www.metware.cn

^7^ http://www.aptbiotech.com/

^8^ http://geneontology.org/

^9^ http://cello.life.nctu.edu.tw/

^10^ https://119.3.166.241/

^11^ https://www.kegg.jp

## Supplementary Material

SUPPLEMENTARY FIGURE S1 KEGG annotation and enrichment of DEGs.

SUPPLEMENTARY FIGURE S2 Total ions current overlaps of the three quality control samples by mass spectrometry detection. **(A)** TIC overlay plot in positive ionization mode. **(B)** TIC overlay plot in negative ionization mode.

SUPPLEMENTARY FIGURE S3 Correlation analysis for all samples and metabolites.

SUPPLEMENTARY FIGURE S4 Heatmap clustering all samples and metabolites.

SUPPLEMENTARY FIGURE S5 KEGG annotation and enrichment of DAMs. **(A)** HN-C vs. GZ-C. **(B)** HN-S vs. GZ-S. **(C)** HN-M vs. GZ-M. **(D)** HN-L vs. GZ-L.

SUPPLEMENTARY FIGURE S6 The quality control (QC) of Proteomics.

SUPPLEMENTARY FIGURE S7 Subcellular localization analysis of all proteins.

SUPPLEMENTARY FIGURE S8 GO analysis of all proteins.

SUPPLEMENTARY FIGURE S9 GO analysis of the DEPs. **(A)** HN-C vs. GZ-C. **(B)** HN-S vs. GZ-S. **(C)** HN-M vs. GZ-M. **(D)** HN-L vs. GZ-L.

SUPPLEMENTARY FIGURE S10 KEGG analysis of the DEPs. **(A)** HN-C vs. GZ-C. **(B)** HN-S vs. GZ-S. **(C)** HN-M vs. GZ-M. **(D)** HN-L vs. GZ-L.

SUPPLEMENTARY FIGURE S11 Domain analysis of the common DEPs.

SUPPLEMENTARY FIGURE S12 GO analysis of the common DEPs.

SUPPLEMENTARY TABLE S1 Phenotypic and physiological characterizations.

SUPPLEMENTARY TABLE S2 The primers of RT-qPCR.

SUPPLEMENTARY TABLE S3 Statistics of transcriptome sequencing results.

SUPPLEMENTARY TABLE S4 Numbers of transcripts, unigenes and new genes from transcriptome sequencing results.

SUPPLEMENTARY TABLE S5 315 secondary metabolite information.

SUPPLEMENTARY TABLE S6 Spectra, peptides, and proteins information.

SUPPLEMENTARY TABLE S7 GO analysis of the DEPs.

SUPPLEMENTARY TABLE S8 KEGG analysis of the DEPs.
