# Supplementary figures and images for: Multiomics Analyses of Two Sorghum Cultivars Reveal the Molecular Mechanism of Salt Tolerance
Source: Front Plant Sci. 2022 May 23;13:886805. doi: 10.3389/fpls.2022.886805 (PMC9168679; doi:10.3389/fpls.2022.886805)

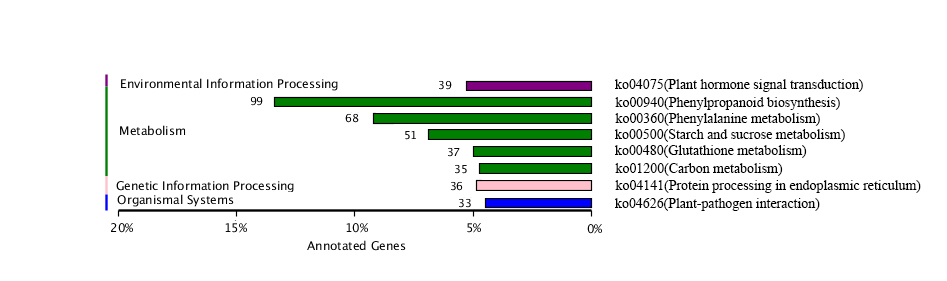

Supplement: SUPPLEMENTARY FIGURE S1 — KEGG annotation and enrichment of DEGs. [file Data_Sheet_1.ZIP › ▓╣│Σ═╝╞1⁄4╝░═╝▒φ/Fig. S1.jpg]

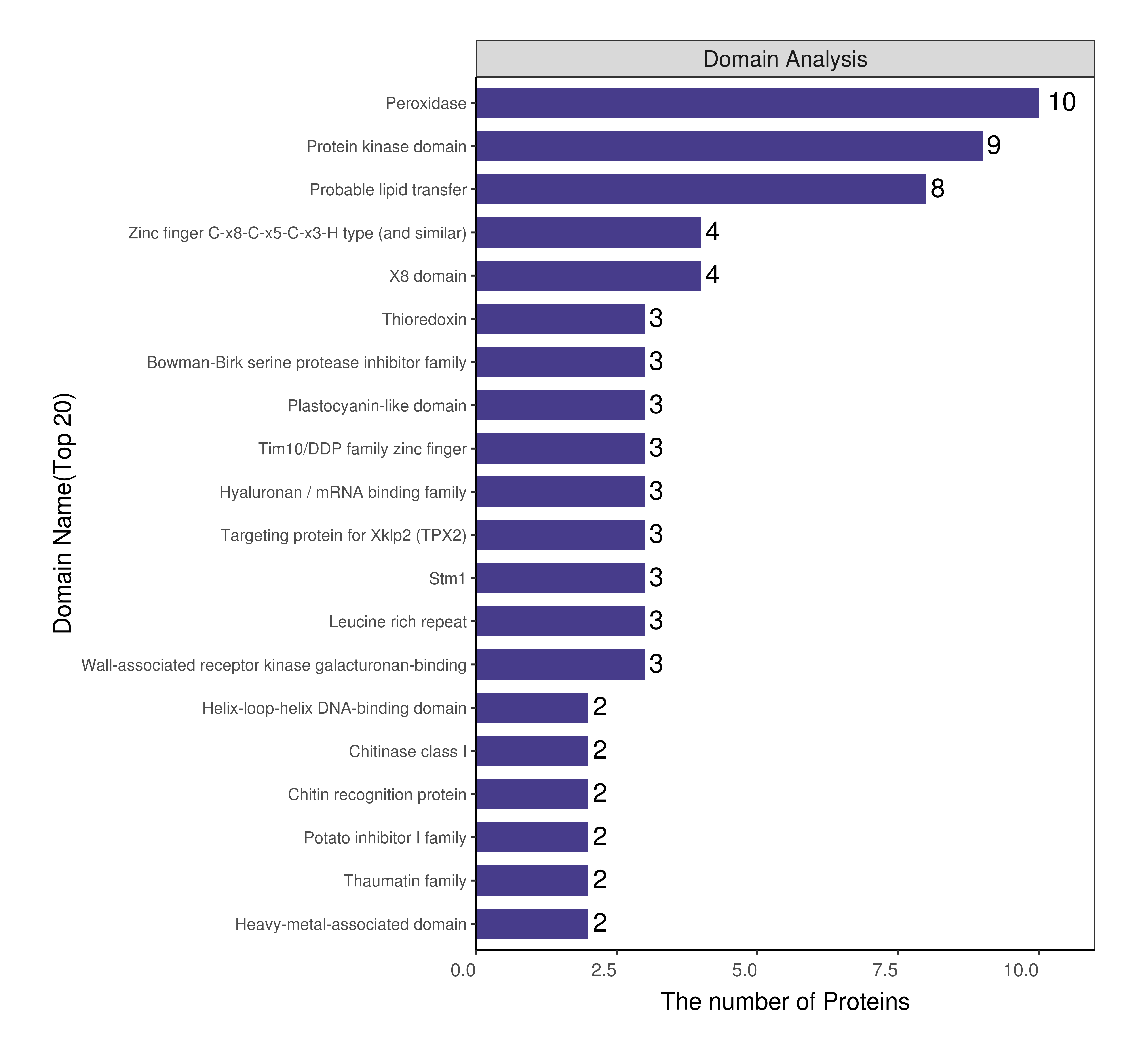

Supplement: SUPPLEMENTARY FIGURE S1 — KEGG annotation and enrichment of DEGs. [file Data_Sheet_1.ZIP › ▓╣│Σ═╝╞1⁄4╝░═╝▒φ/Fig. S11.png]

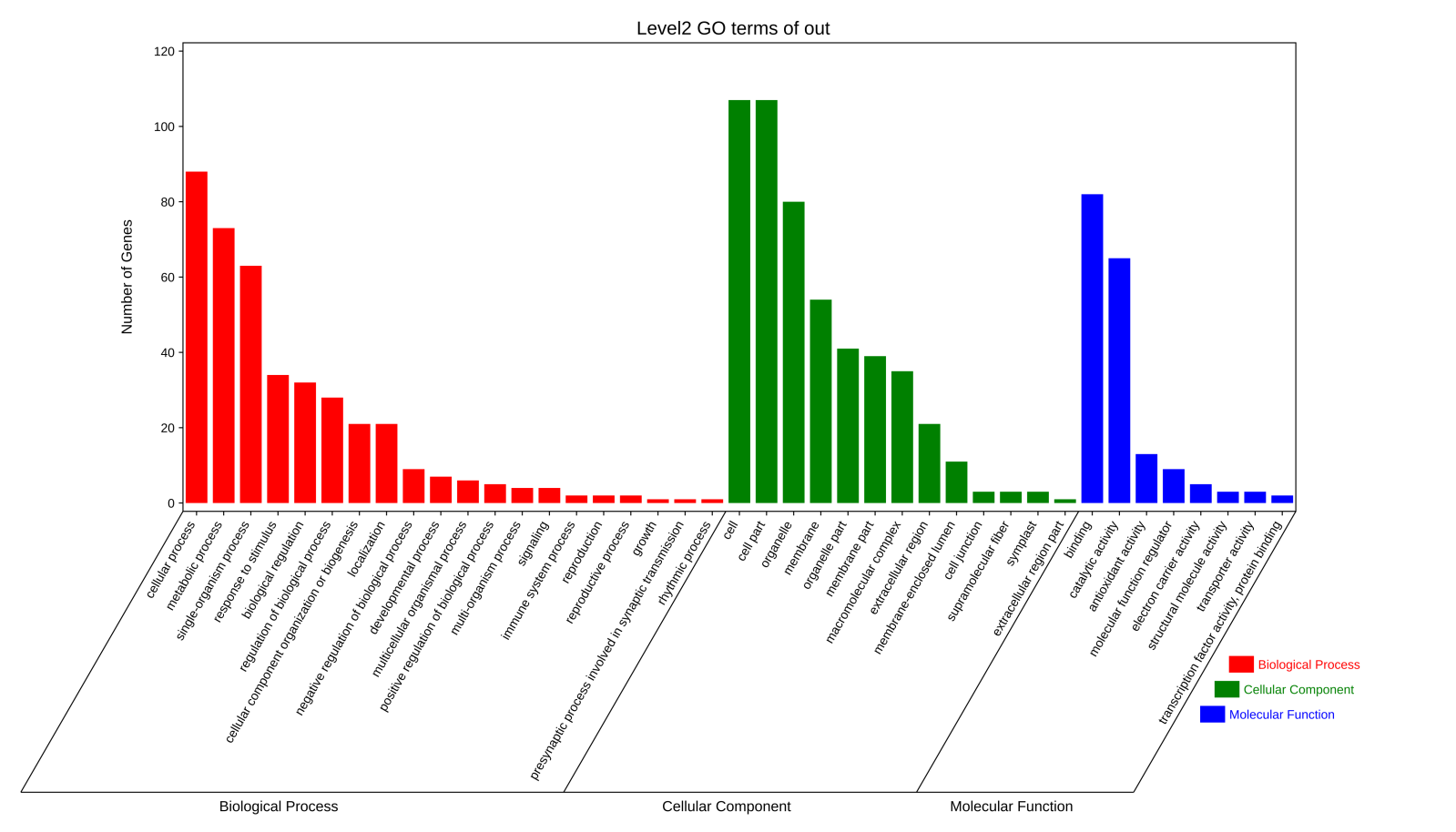

Supplement: SUPPLEMENTARY FIGURE S1 — KEGG annotation and enrichment of DEGs. [file Data_Sheet_1.ZIP › ▓╣│Σ═╝╞1⁄4╝░═╝▒φ/Fig. S12.png]

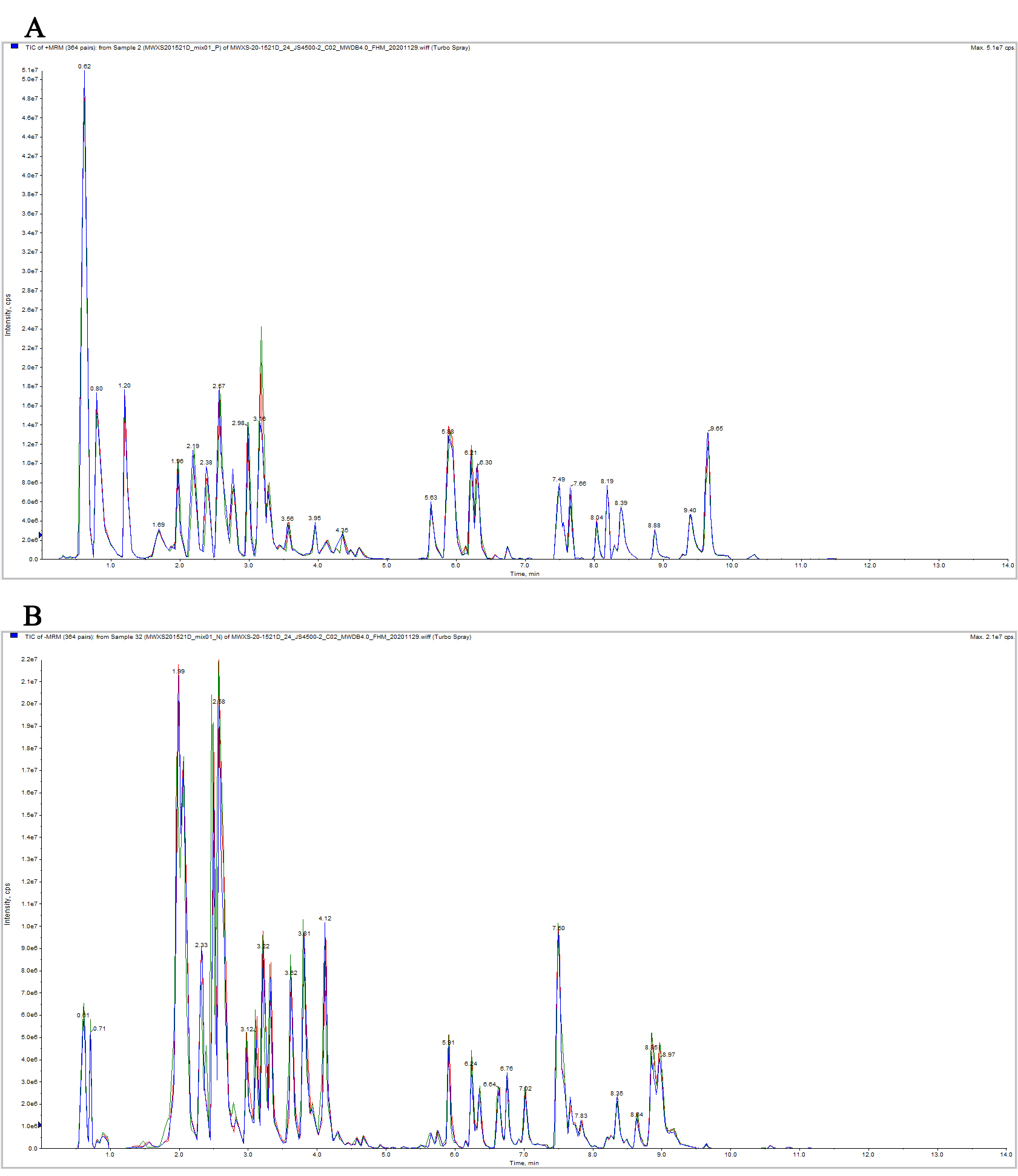

Supplement: SUPPLEMENTARY FIGURE S1 — KEGG annotation and enrichment of DEGs. [file Data_Sheet_1.ZIP › ▓╣│Σ═╝╞1⁄4╝░═╝▒φ/Fig. S2.jpg]

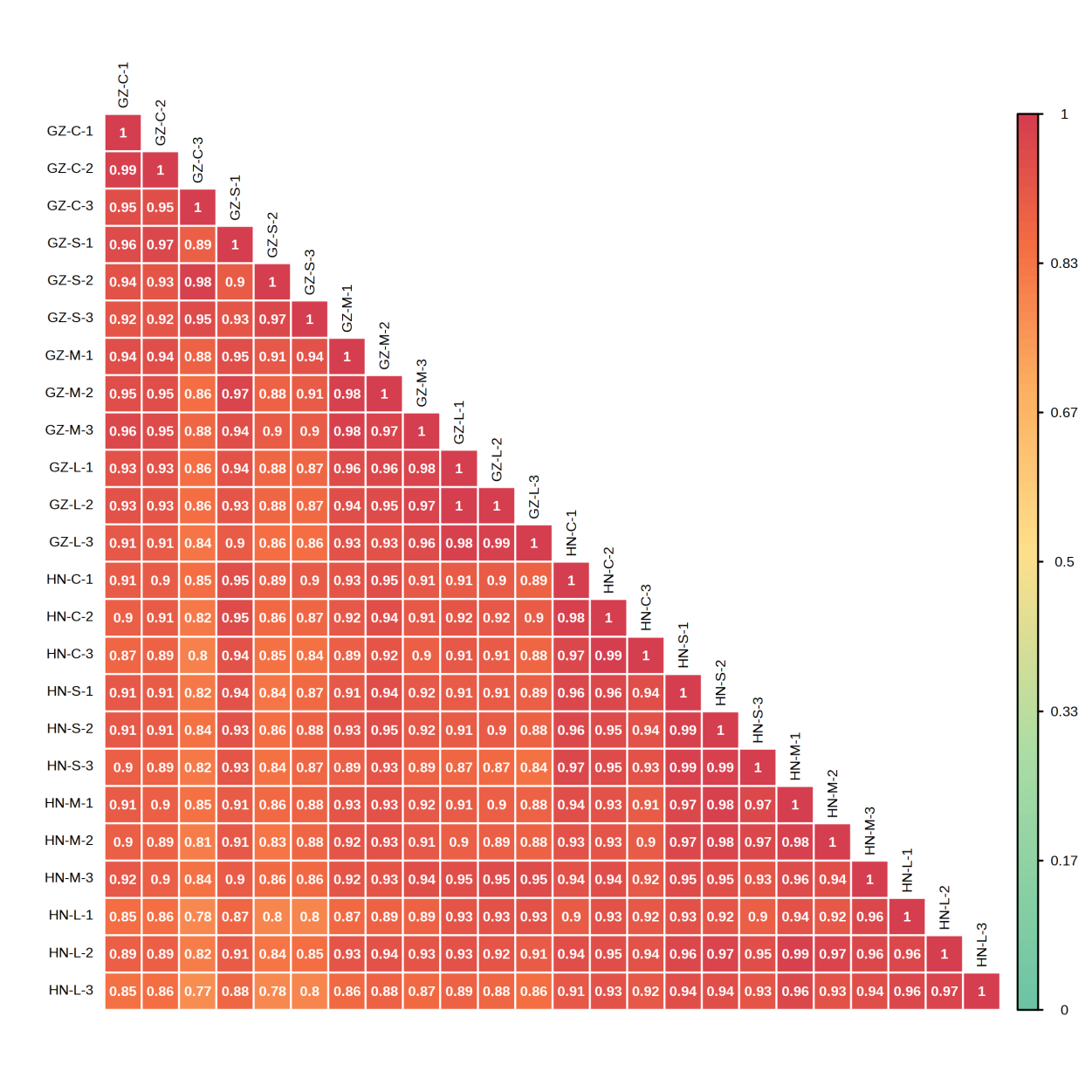

Supplement: SUPPLEMENTARY FIGURE S1 — KEGG annotation and enrichment of DEGs. [file Data_Sheet_1.ZIP › ▓╣│Σ═╝╞1⁄4╝░═╝▒φ/Fig. S3.png]

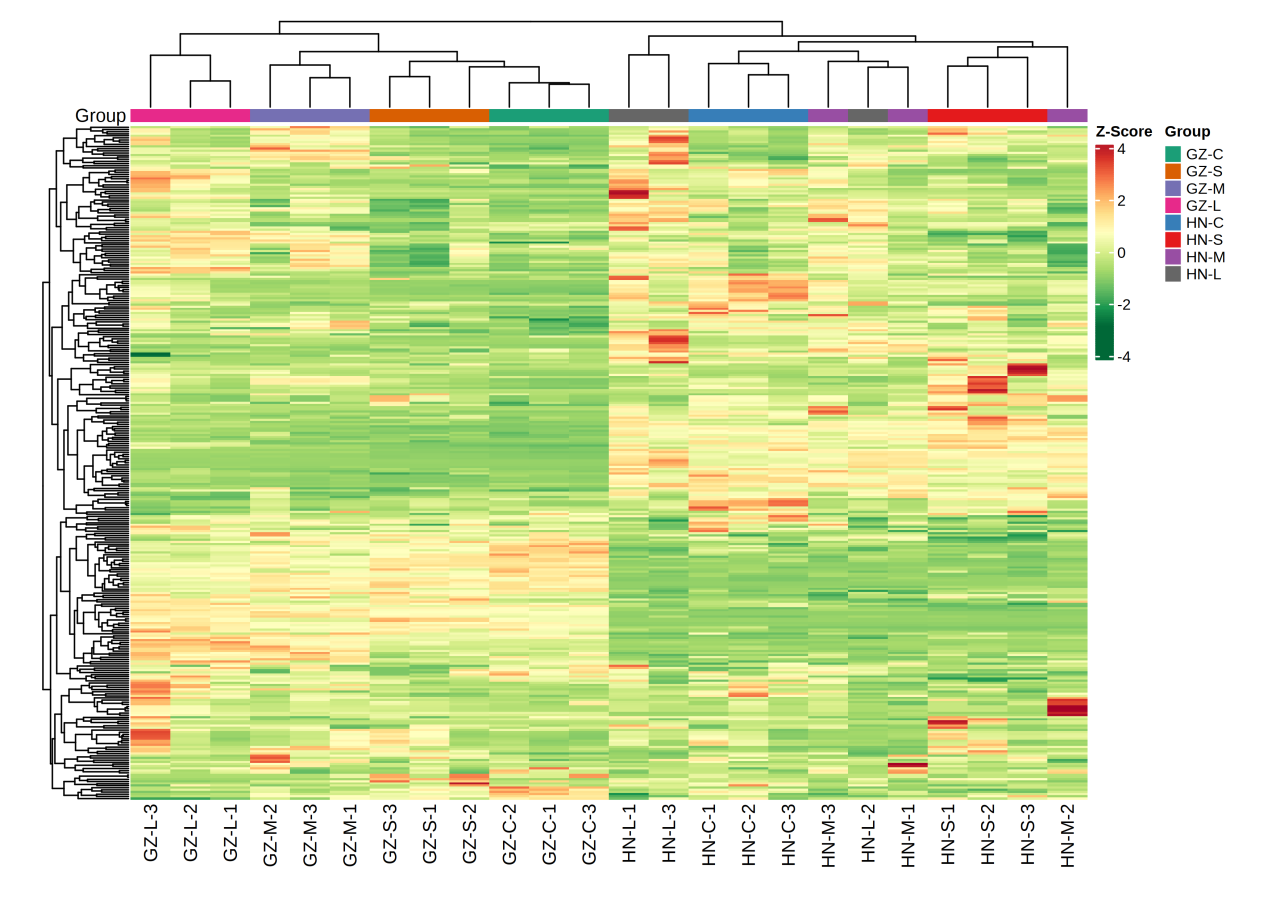

Supplement: SUPPLEMENTARY FIGURE S1 — KEGG annotation and enrichment of DEGs. [file Data_Sheet_1.ZIP › ▓╣│Σ═╝╞1⁄4╝░═╝▒φ/Fig. S4.png]

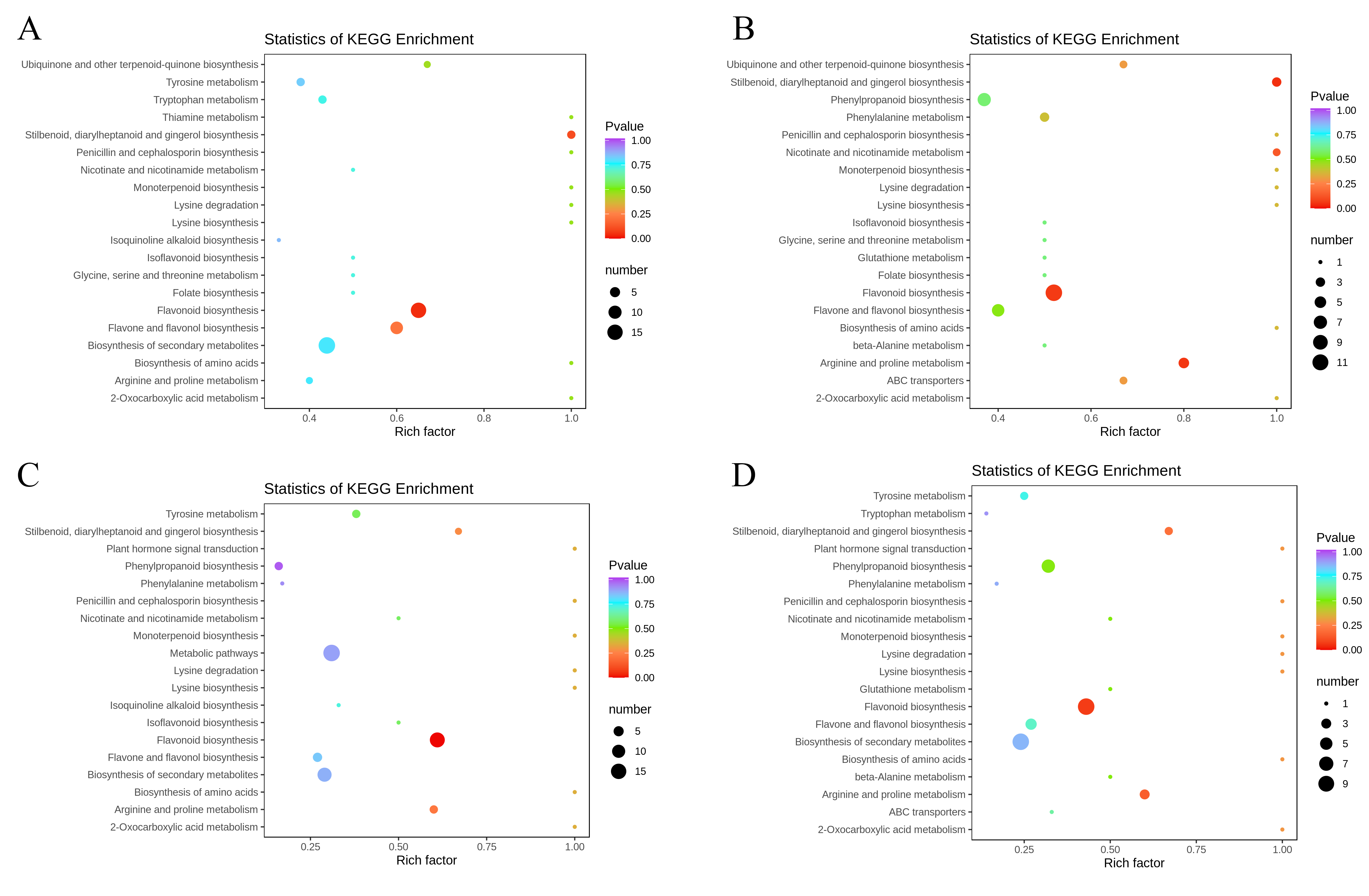

Supplement: SUPPLEMENTARY FIGURE S1 — KEGG annotation and enrichment of DEGs. [file Data_Sheet_1.ZIP › ▓╣│Σ═╝╞1⁄4╝░═╝▒φ/Fig. S5.jpg]

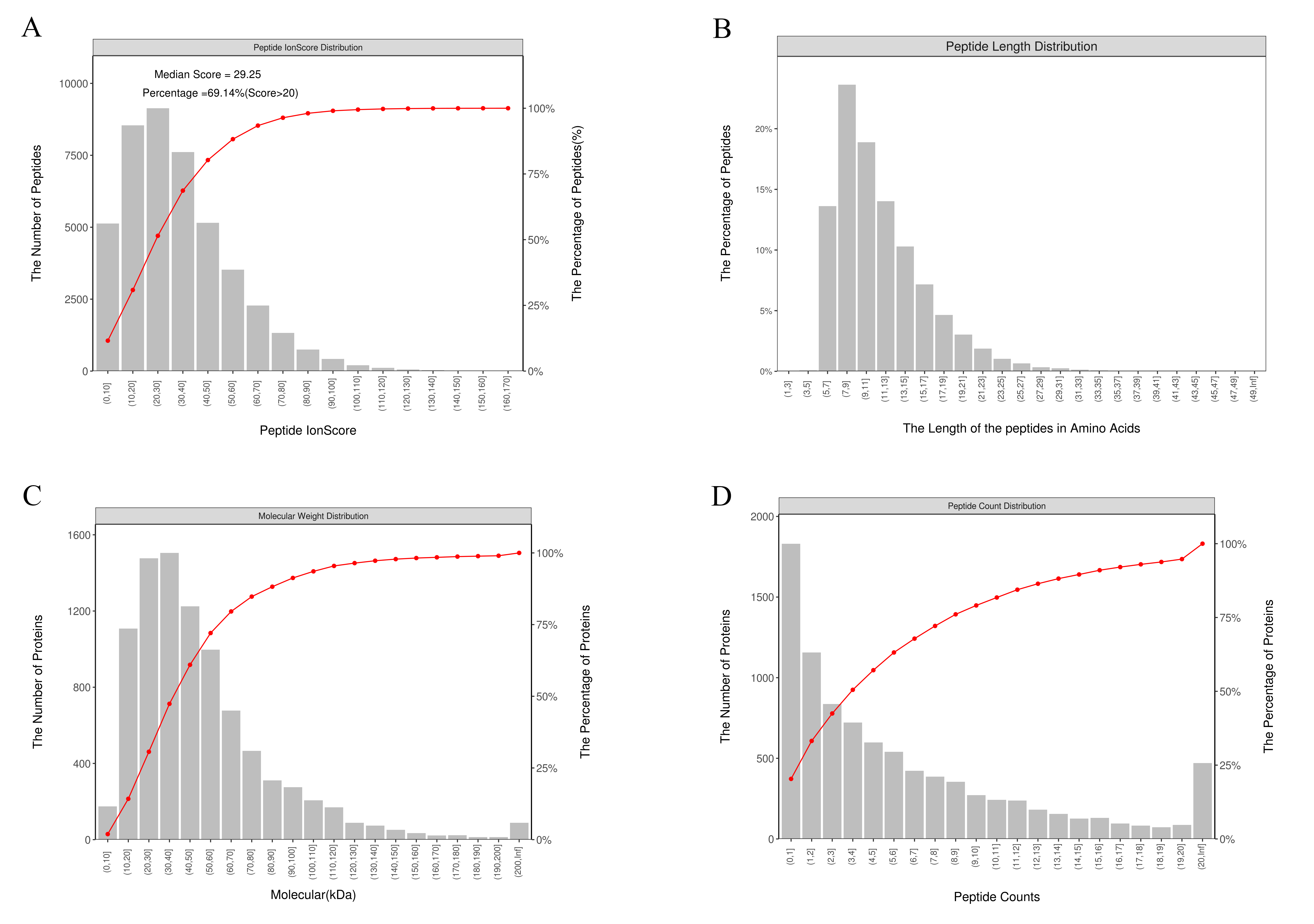

Supplement: SUPPLEMENTARY FIGURE S1 — KEGG annotation and enrichment of DEGs. [file Data_Sheet_1.ZIP › ▓╣│Σ═╝╞1⁄4╝░═╝▒φ/Fig. S6.jpg]

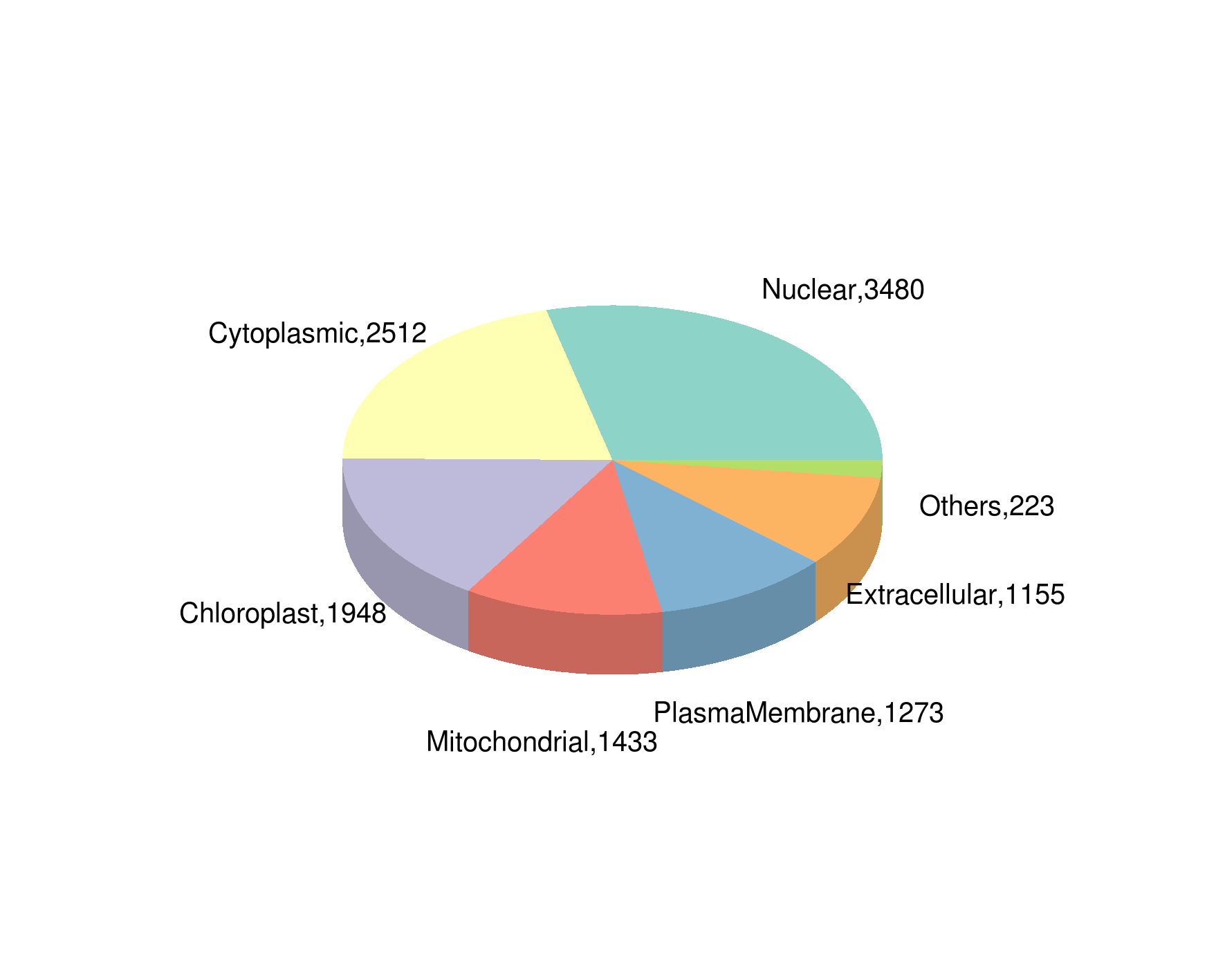

Supplement: SUPPLEMENTARY FIGURE S1 — KEGG annotation and enrichment of DEGs. [file Data_Sheet_1.ZIP › ▓╣│Σ═╝╞1⁄4╝░═╝▒φ/Fig. S7.png]

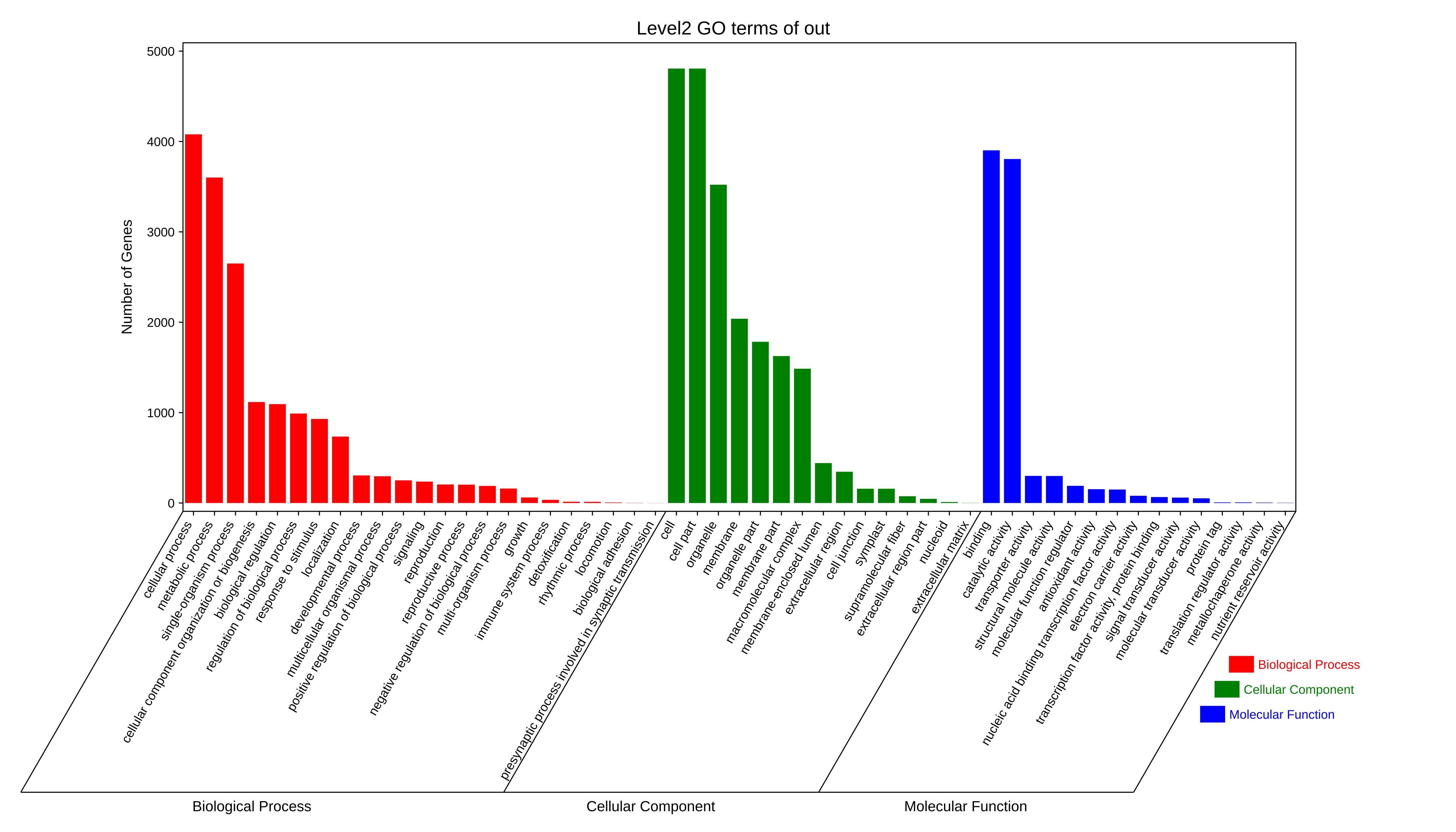

Supplement: SUPPLEMENTARY FIGURE S1 — KEGG annotation and enrichment of DEGs. [file Data_Sheet_1.ZIP › ▓╣│Σ═╝╞1⁄4╝░═╝▒φ/Fig. S8.png]
